# Supplementary material for: Suppressing phase disproportionation in quasi-2D perovskite light-emitting diodes
Source: Nat Commun. 2023 Jan 25;14:397. doi: 10.1038/s41467-023-36118-7 (PMC9873927; doi:10.1038/s41467-023-36118-7)
Supplement: Supplementary file 1 — Supplementary Information [file 41467_2023_36118_MOESM1_ESM.pdf]

Supplementary Information for

Suppressing Phase Disproportionation in Quasi-2D Perovskite Light-Emitting Diodes

Kang Wang, Zih-Yu Lin, Zihan Zhang, Linrui Jin, Ke Ma, Aidan H. Coffey, Harindi R. Atapattu, Yao Gao, Jee Yung Park, Zitang Wei, Blake P. Finkenauer, Chenhui Zhu, Xiangeng Meng, Sarah N. Chowdhury, Zhaoyang Chen, Tauguy Terlier, Thi-Hoai Do, Yan Yao, Kenneth R. Graham, Alexandra Boltasseva, Tzung-Fang Guo, Libai Huang, Hanwei Gao, Brett M. Savoie, Letian Dou*

*Corresponding author. E-mail: dou10@purdue.edu (L.D.)

This file contains

- Supplementary Figures 1 – 51
- Supplementary Tables 1 – 5
- ^{1}H, ^{13}C NMR and HR-MS spectra
- References

1. Materials synthesis

All reactions sensitive to air and water were performed in an inert (argon) atmosphere using a Schlenk line setup and tubes. All chemical reagents and solvents were purchased from Sigma-Aldrich and used as received.

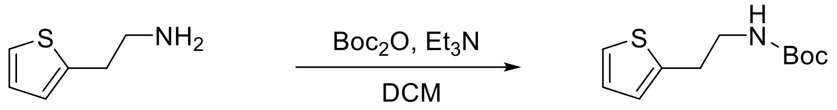

1

***tert*-butyl (2-(thiophen-2-yl)ethyl)carbamate (1)**: 2-(thiophen-2-yl)ethyl-1-amine (15 g, 117.9 mmol) and 150 mL dry dichloromethane (DCM) were added to a dried 250 mL round bottom flask. Then, triethylamine (24.65 mL, 176.8 mmol) was added to the solution. Di-*tert*-butyl dicarbonate (30.88 g, 141.15 mmol) was then added portion-wise which caused bubbles to evolve from the solution. After stirring at room temperature for 4 hours, the reaction mixture was washed with water. The organic layer was collected, dried over Mg_2SO_4 and the solid was filtered. The solvent was removed under reduced pressure to provide a yellow oil (98 %), which was directly used for the next step without further purification. ^{1}H NMR (400 MHz, Chloroform-*d*) δ 7.17-7.14 (m, 1H), 6.94 (dd, J = 5.2, 3.4 Hz, 1H), 6.84-6.82 (m, 1H), 4.65 (s, 1H), 3.40 (d, J = 6.5 Hz, 2H), 3.01 (t, J = 6.7 Hz, 2H), 1.44 (s, 9H).

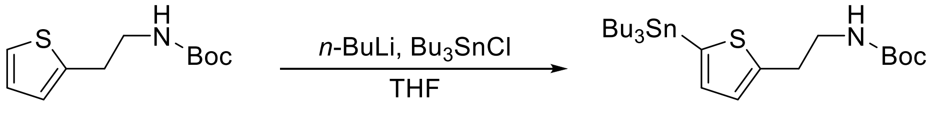

2

***tert*-butyl (2-(5-(tributylstannyl)thiophen-2-yl)ethyl)carbamate (2)**: 10.5 g of **1** (46.19 mmol) was dissolved in 250 mL dry THF and a 1.6 M solution of *n*-BuLi in hexane (63.46 mL, 101.54 mmol) was added dropwise at 0 ºC. The mixture was stirred for 3 hour at 0 ºC and then tri(*n*-butyl)stannyl chloride (28.06 mL, 103.83 mmol) was added. The temperature was raised to room temperature and the solution was stirred for 2 hours. Afterwards the solution mixture was poured into water and extracted with DCM. The organic phase was washed with water and brine and dried over Mg_2SO_4. The solvent was removed under reduced pressure to produce yellowish oil and directly used for the next step without further purification. ^{1}H NMR (400 MHz, Chloroform-*d*) δ 7.00 (d, J = 3.1 Hz, 1H), 6.94 (d, J = 3.2 Hz, 1H), 4.66 (s, 1H), 3.41 (d, J = 7.2 Hz, 2H), 3.05 (t, J = 6.7 Hz, 2H), 1.59 – 1.50 (m, 6H), 1.42 (s, 9H), 1.37 – 1.29 (m, 6H), 1.11 – 1.01 (m, 6H), 0.97 – 0.81 (m, 9H).

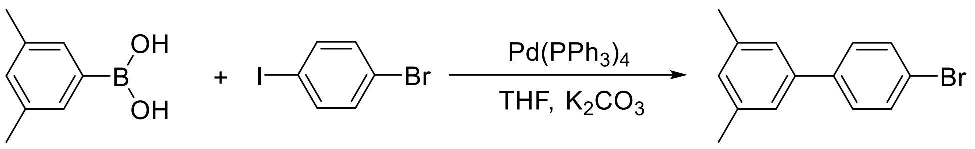

3'

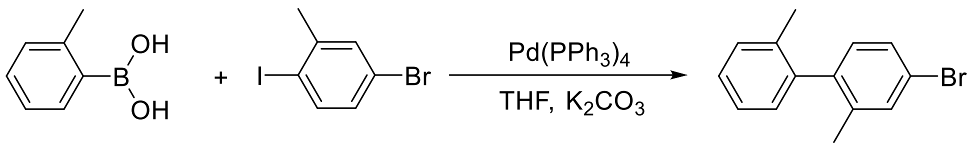

3

4'-bromo-3,5-dimethyl-1,1'-biphenyl (**3'**): (3,5-dimethylphenyl)boronic acid (3.30 g, 22 mmol), 1-bromo-4-iodobenzene (5.66 g, 20 mmol), $Pd(PPh_3)_4$ (1.15 g, 1 mmol), and K_2CO_3 (22.11 g, 160 mmol) were added into a dry two-neck round bottom flask. After replacing the air with argon, THF (200 mL) and DI water (80 mL) were added via syringe. Then the mixture was stirred and refluxed overnight under Ar. After cooling to room temperature, water was added, and the mixture was extracted with DCM for 3 times. The organic layers were combined, washed with water and brine, and dried over Mg_2SO_4. The solids were filtered, and the filtrate was concentrated via rotary evaporation. The crude product was further purified by column chromatography with hexanes as eluent. The product was collected as white solid (3.7 g, 71%). ^{1}H NMR (400 MHz, Chloroform-*d*) δ 7.53 (d, J = 8.2 Hz, 2H), 7.43 (d, J = 8.2 Hz, 2H), 7.16 (s, 2H), 7.01 (s, 1H), 2.37 (s, 6H).

4-bromo-2,2'-dimethyl-1,1'-biphenyl (**3**): *o*-tolylboronic acid (2.99 g, 22 mmol), 4-bromo-1-iodo-2-methylbenzene (5.94 g, 20 mmol), $Pd(PPh_3)_4$ (1.15 g, 1 mmol), and K_2CO_3 (22.11 g, 160 mmol) were added into a dry two-neck round bottom flask. After replacing the air with argon, THF (200 mL) and DI water (80 mL) were added via syringe. Then the mixture was stirred and fluxed overnight under Ar. After cooling to room temperature, water was added, and the mixture was extracted with DCM for 3 times. The organic layers were combined, washed with water and brine, and dried over Mg_2SO_4. The solids were filtered, and the filtrate was concentrated via rotary evaporation. The crude product was further purified by column chromatography with hexanes as eluent. The product was collected as an oil (4.0 g, 76%). ^{1}H NMR (400 MHz, Chloroform-*d*) δ 7.43 (d, J = 2.1 Hz, 1H), 7.36 (dd, J = 8.2, 2.2 Hz, 1H), 7.29 – 7.26 (m, 2H), 7.25 – 7.20 (m, 1H), 7.06 (d, J = 7.2 Hz, 1H), 6.98 (d, J = 8.1 Hz, 1H), 2.05 (s, 3H), 2.04 (s, 3H).

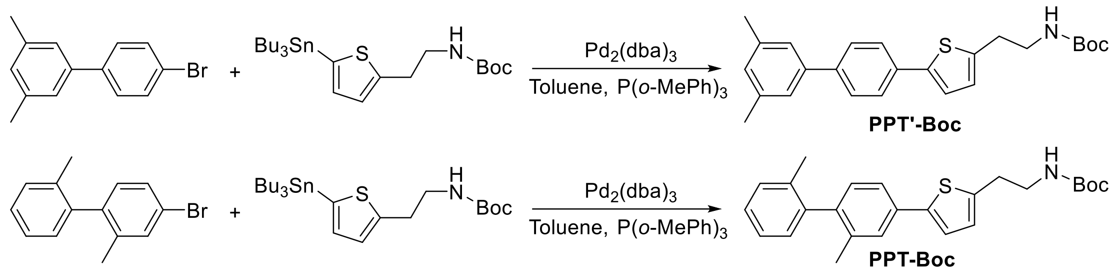

***tert*-butyl(2-(5-(3',5'-dimethyl-[1,1'-biphenyl]-4-yl)thiophen-2-yl)ethyl)carbamate** (**PPT'-Boc**): 1.31 g of **3'** (5 mmol), 2.58 g of **2** (5 mmol), $Pd_2(dba)_3$ (916 mg, 0.1 mmol), and P(*o*-MePh)$_3$ (122 mg, 0.4 mmol) were added in a two-neck dry round bottom flask. After replacing the air with argon, anhydrous toluene (50 mL) was added via syringe. The mixture was stirred at 100 ^{o}C overnight. After cooling to room temperature, water was added, and the mixture was extracted with DCM for 3 times. The organic layers were combined, washed with water and brine, and dried over Mg_2SO_4. The solids were filtered, and the filtrate was concentrated via rotary evaporation. The crude product was further purified by column chromatography with ethyl acetate: hexane (1:3) as the eluent. The product was collected as a yellowish oil (1.1 g, 54%). ^{1}H NMR (400 MHz, Chloroform-*d*) δ 7.64 - 7.53 (m, 4H), 7.22 (d, *J* = 1.5 Hz, 2H), 7.18 (d, *J* = 3.5 Hz, 1H), 7.00 (s, 1H), 6.81 (d, *J* = 3.8 Hz, 1H), 4.70 (s, 1H), 3.44 (d, *J* = 7.1 Hz, 2H), 3.02 (t, *J* = 6.7 Hz, 2H), 2.38 (s, 6H), 1.45 (s, 9H).

***tert*-butyl (2-(5-(2,2'-dimethyl-[1,1'-biphenyl]-4-yl)thiophen-2-yl)ethyl)carbamate** (**PPT-Boc**): 1.31 g of **3** (5 mmol), 2.58 g of **2** (5 mmol), $Pd_2(dba)_3$ (916 mg, 0.1 mmol), and P(*o*-MePh)$_3$ (122 mg, 0.4 mmol) were added in a two-neck dry round bottom flask. After replacing the air with argon, anhydrous toluene (50 mL) was added via syringe. The mixture was stirred at 100 ^{o}C overnight. After cooling to room temperature, water was added, and the mixture was extracted with DCM for 3 times. The organic layers were combined, washed with water and brine, and dried over Mg_2SO_4. The solids were filtered, and the filtrate was concentrated via rotary evaporation. The crude product was further purified by column chromatography with ethyl acetate: hexane (1:3) as the eluent. The product was collected as a yellowish oil (1.2 g, 59%). ^{1}H NMR (400 MHz, Chloroform-*d*) δ 7.46 (d, *J* = 2.0 Hz, 1H), 7.42 (dd, *J* = 7.8, 2.0 Hz, 1H), 7.29 – 7.26 (m, 2H), 7.22 (dt, *J* = 5.9, 3.4 Hz, 1H), 7.17 (d, *J* = 3.7 Hz, 1H), 7.13 – 7.08 (m, 2H), 6.81 (d, *J* = 3.5 Hz, 1H), 4.70 (s, 1H), 3.44 (d, *J* = 7.1 Hz, 2H), 3.02 (t, *J* = 6.7 Hz, 2H), 1.45 (s, 9H).

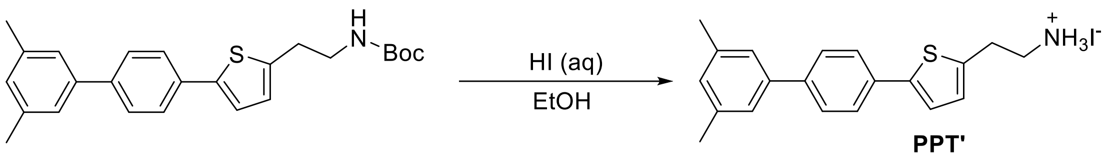

PPT'

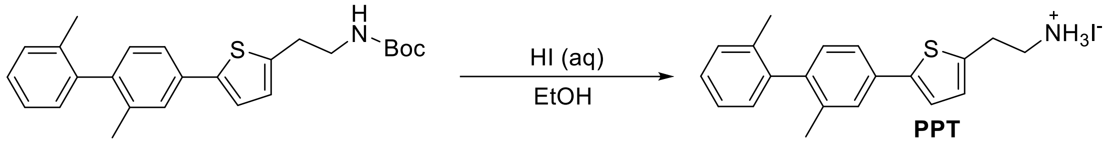

PPT

2-(5-(3',5'-dimethyl-[1,1'-biphenyl]-4-yl)thiophen-2-yl)ethyl-1-ammonium iodide (**PPT'**): PTT'-Boc (1.0 g, 2.4 mmol) was dissolved in 24 mL ethanol, and 2.0 equiv aqueous HI solution (634 μL, 37 wt%, 4.8 mmol) was then added. The mixture was refluxed for 2 h to cleave the Boc protecting group, then cooled to the room temperature. Next, the solvents were removed under reduced pressure. After adding diethyl ether to the residue, the solid products were collected by filtration, which was washed several times with diethyl ether. The products were dried under vacuum as white powder (948 mg, 91 %). ^{1}H NMR (400 MHz, DMSO-d_6) δ 7.80 (s, 3H), 7.66 (s, 4H), 7.43 (d, J = 3.7 Hz, 1H), 7.27 (d, J = 1.6 Hz, 2H), 6.99 (q, J = 3.7 Hz, 2H), 3.10 (q, J = 3.2 Hz, 4H), 2.32 (s, 6H). ^{13}C NMR (100 MHz, DMSO-d_6) δ 142.04, 139.64, 139.11, 138.33, 132.95, 129.39, 127.94, 127.62, 125.85, 124.62, 124.12, 27.90, 21.40. HR-MS (ESI) Expected 308.1468 $[M – I]^+$ Observed 308.1467.

2-(5-(2,2'-dimethyl-[1,1'-biphenyl]-4-yl)thiophen-2-yl)ethyl-1-ammonium iodide (**PPT**): PTT-Boc (1.0 g, 2.4 mmol) was dissolved in 24 mL ethanol, and 2.0 equiv aqueous HI solution (634 μL, 37 wt%, 4.8 mmol) was then added. The mixture was refluxed for 2 h to cleave the Boc protecting group, then cooled to the room temperature. Next, the solvents were removed under reduced pressure. After adding diethyl ether to the residue, the solid products were collected by filtration, which was washed several times with diethyl ether. The products were dried under vacuum as white powder (1.0 g, 95 %). ^{1}H NMR (400 MHz, DMSO-d_6) δ 7.79 (s, 3H), 7.53 (d, J = 2.0 Hz, 1H), 7.45 (dd, J = 7.9, 2.0 Hz, 1H), 7.40 (d, J = 3.7 Hz, 1H), 7.32 – 7.20 (m, 3H), 7.11 – 7.04 (m, 2H), 6.99 (d, J = 3.7 Hz, 1H), 3.09 (q, J = 3.8 Hz, 4H), 2.01 (d, J = 4.2 Hz, 6H). ^{13}C NMR (100 MHz, DMSO-d_6) δ 142.28, 140.80, 140.72, 138.89, 136.41, 135.49, 133.00, 130.26, 130.21, 129.34, 127.79, 126.83, 126.13, 123.96, 122.91, 27.90, 19.92, 19.88. HR-MS (ESI) Expected 308.1468 $[M – I]^+$ Observed 308.1465.

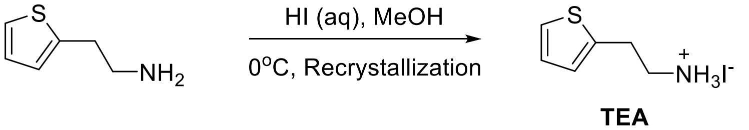

TEA

2-(thiophen-2-yl)ethyl-1-aminium iodide (TEA): 2-(thiophen-2-yl)ethyl-1-amine (1.27 g, 10 mmol) and 100 mL dry methanol were added to a dried 250 mL round bottom flask. The above solution was stirred at 0 °C and 1.2 equiv aqueous HI solution (1584 μL, 37 wt%, 12 mmol) was then added. After stirring for 2 hours, the solvents were partially removed under reduced pressure to obtain a hot saturated solution. The residue was poured into large amount of diethyl ether and sit still in refrigerator overnight for fully recrystallization. The solid products were collected by filtration, which was washed at least 5 times with cold diethyl ether. The products were dried overnight under vacuum to yield white powder (2.09 g, 82 %). ^{1}H NMR (400 MHz, DMSO-d_6) δ 7.69 (s, 3H), 7.40 (d, J = 5.0 Hz, 1H), 7.02 – 6.93 (m, 2H), 3.05 (s, 4H).

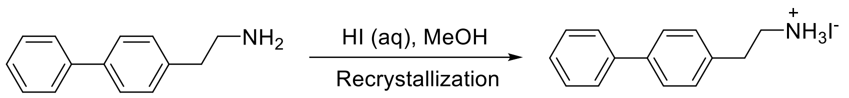

2P

2-([1,1'-biphenyl]-4-yl)ethyl-1-ammonium iodide (2P): 1.97 g of 2-([1,1'-biphenyl]-4-yl)ethyl-1-amine (10 mmol) and 100 mL dry methanol were added to a dried 250 mL round bottom flask. The above solution was stirred at 60 °C and 1.2 equiv aqueous HI solution (1584 μL, 37 wt%, 12 mmol) was then added. After stirring for 4 hours, the solvents were partially removed under reduced pressure to obtain a hot saturated solution. The residue was poured into large amount of toluene and sit still in refrigerator overnight for fully recrystallization. The solid products were collected by filtration, which was washed at least 5 times with cold diethyl ether. The products were dried overnight under vacuum to yield white powder (1.66, 51%). ^{1}H NMR (400 MHz, DMSO-d_6) δ 7.85 (s, 3H), 7.62 (m, 4H), 7.44 (t, J = 7.6 Hz, 2H), 7.34 (m, 3H), 3.07 (t, J = 7.9 Hz, 2H), 2.90 (t, J = 7.9 Hz, 2H).

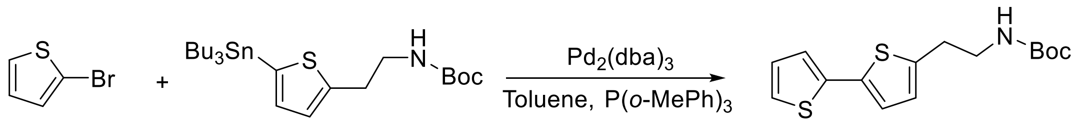

2T-Boc

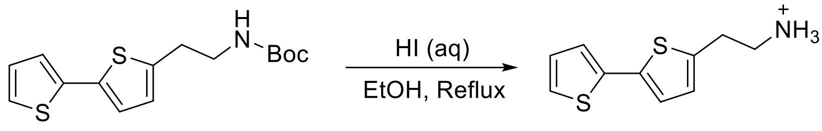

2T

***tert*-butyl (2-([2,2'-bithiophen]-5-yl)ethyl)carbamate (2T-Boc)[1]:** 815 mg of 2-bromothiophene (5 mmol), 2.58 g of **2** (5 mmol), $Pd_2(dba)_3$ (916 mg, 0.1 mmol),

and $P(o\text{-}MePh)_3$ (122 mg, 0.4 mmol) were added in a two-neck dry round bottom flask. After replacing the air with argon, anhydrous toluene (50 mL) was added via syringe. The mixture was stirred at 100 °C overnight. After cooling to room temperature, water was added, and the mixture was extracted with DCM 3 times. The organic layers were combined, washed with water and brine, and dried over Mg_2SO_4. The solids were filtered, and the filtrate was concentrated via rotary evaporation. The crude product was further purified by column chromatography with ethyl acetate: hexane (1:3) as the eluent. The product was collected as a yellowish oil (928 mg, 60%).

2-([2,2'-bithiophen]-5-yl)ethyl-1-ammonium iodide (2T)[1]: 2T-Boc (928 mg, 3.0 mmol) was dissolved in 30 mL ethanol, and 2.0 equiv aqueous HI solution (793 μL, 37 wt%, 6.0 mmol) was then added. The mixture was refluxed for 2 h to cleave the Boc protecting group, then cooled to room temperature. Next, the solvents were removed under reduced pressure. After adding diethyl ether to the residue, the solid products were collected by filtration, which was washed several times with diethyl ether. The products were dried under vacuum as white powder (890 mg, 88 %).

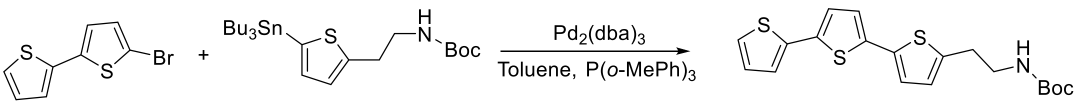

3T-Boc

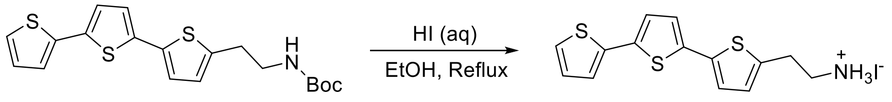

3T

***tert*-butyl (2-([2,2':5',2''-terthiophen]-5-yl)ethyl)carbamate (3T-Boc)[1]:** 1.22 g of 5-bromo-2,2'-bithiophene (5 mmol), 2.58 g of **2** (5 mmol), $Pd_2(dba)_3$ (916 mg, 0.1 mmol), and $P(o\text{-}MePh)_3$ (122 mg, 0.4 mmol) were added in a two-neck dry round bottom flask. After replacing the air with argon, anhydrous toluene (50 mL) was added via syringe. The mixture was stirred at 100 °C overnight. After cooling to room temperature, water was added, and the mixture was extracted with DCM 3 times. The organic layers were combined, washed with water and brine, and dried over Mg_2SO_4. The solids were filtered, and the filtrate was concentrated via rotary evaporation. The crude product was further purified by column chromatography with ethyl acetate: hexane (1:3) as the eluent. The product was collected as a yellowish oil (1.27 g, 65%).

2-([2,2':5',2''-terthiophen]-5-yl)ethyl-1-aminium iodide (3T)[1]: (1.27 g, 3.25 mmol) was dissolved in 30 mL ethanol, and 2.0 equiv aqueous HI solution (859 μL, 37 wt%,

6.5 mmol) was then added. The mixture was refluxed for 2 h to cleave the Boc protecting group, then cooled to room temperature. Next, the solvents were removed under reduced pressure. After adding diethyl ether to the residue, the solid products were collected by filtration, which was washed several times with diethyl ether. The products were dried under vacuum as white powder (1.14 g, 84 %).

2. Supplementary figures and related discussions

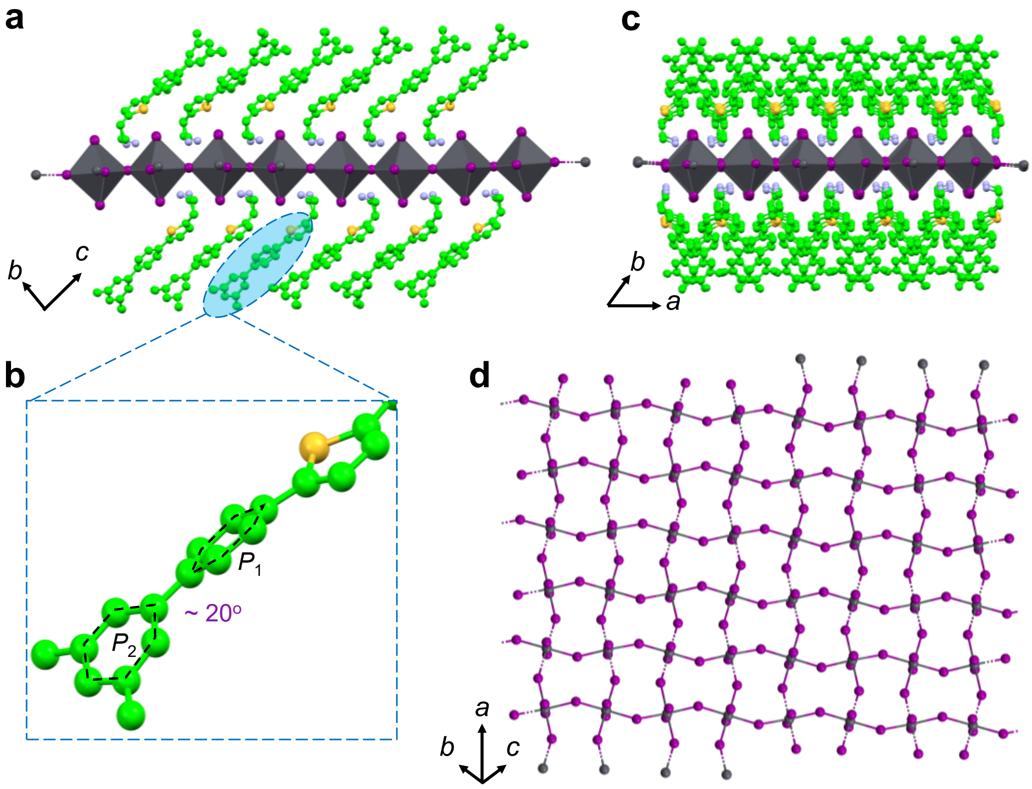

Supplementary Fig. 1 | Depictions of the single crystal X-ray diffraction results for (PPT')$_2$PbI$_4$. a, View along *a* axis. **b,** Zoomed-in view of dihedral angles between two phenyl rings in ligand. **c,** View along *c* axis. **d,** View along *a* axis omitting organic ligands. Purple dots represent iodine atoms; dark grey dots represent lead atoms; green dots represent carbon atoms; yellow dots represent sulfur atoms; blue dots represent nitrogen atoms. H atoms are omitted for clarity.

Based on the single crystal structure, the two methyl groups on the 3'- and 5'-positions tethered to phenyl rings of the PPT' ligands experienced a large steric hindrance, which leads to a dihedral angle of ~ 20° between the two phenyl rings, thus enhancing the cross-sectional area and the effective bulkiness of the ligand. These are expected to block the ion diffusion channel between organic ligands in 2D perovskite.

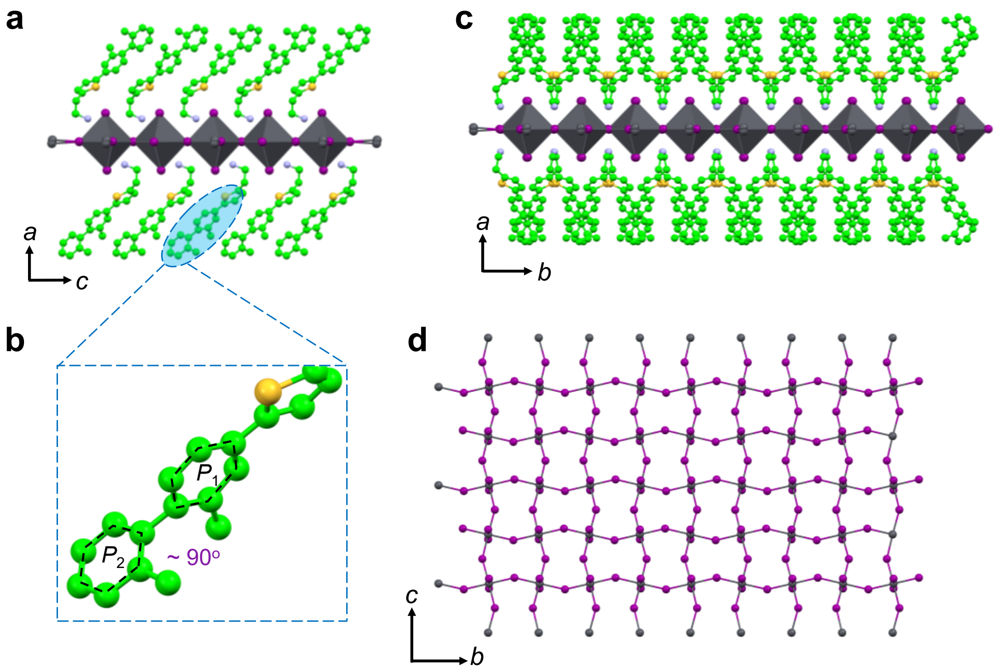

Supplementary Fig. 2 | Depictions of the single crystal X-ray diffraction results for $(PPT)_2PbI_4$. a, View along *b* axis. **b,** Zoomed-in view of dihedral angles between two phenyl rings in ligand. **c,** View along *c* axis. **d,** View along *a* axis omitting organic ligands. Purple dots represent iodine atoms; dark grey dots represent lead atoms; green dots represent carbon atoms; yellow dots represent sulfur atoms; blue dots represent nitrogen atoms. The disorders and H atoms are all omitted for clarity.

Based on the single crystal structure, the two methyl groups on the 2- and 2'- positions tethered to phenyl rings of the PPT ligands experienced a larger steric hindrance so that the planarity is broken, which leads increased dihedral angle (nearly 90°) between the two phenyl rings, thus further enhancing the cross-sectional area and the effective bulkiness of the ligand. These are expected to better block the ion diffusion channel between organic ligands in 2D perovskite. In addition, the less planar structure may help to decrease the intermolecular interaction and improve their solubility in solvents for easier processing.

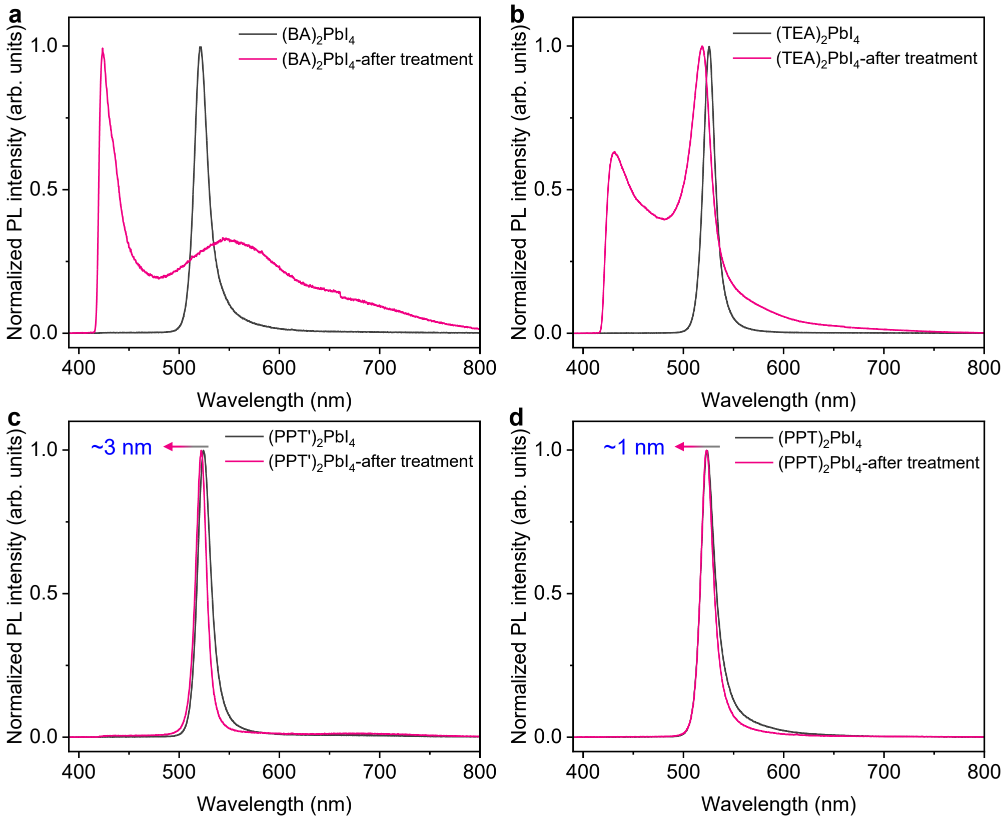

Supplementary Fig. 3 | PL spectra of 2D perovskite thin films before (black) and after (pink) HBr vapor treatment. a, $(BA)_2PbI_4$; **b,** $(TEA)_2PbI_4$; **c,** $(PPT')_2PbI_4$; and **d,** $(PPT)_2PbI_4$. Before treatment, all films show green emissions around 520 nm. After treatment, the PL emission of BA sample completely shift to purple region around 420 nm accompanied by a broad trap-state emission around 550 nm (Supplementary Fig. 3a), suggesting that I was totally substituted by Br. While TEA sample exhibits a mixed purple and green emissions around 433 and 518 nm (Supplementary Fig. 3b) after treatment, indicating a partial substitution of I by Br. Interestingly, the PPT' and PPT samples did not show noticeable emission at purple region even after treatment, demonstrating the suppression of ion penetration through the ligand layer into the perovskite layer. In addition, $(PPT)_2PbI_4$ film demonstrated reduced blue shift (~1 nm) compared to $(PPT')_2PbI_4$ film (~3 nm) after treatment (Supplementary Fig. 3c-d), suggesting a slightly better protection from ion penetration by PPT ligand.

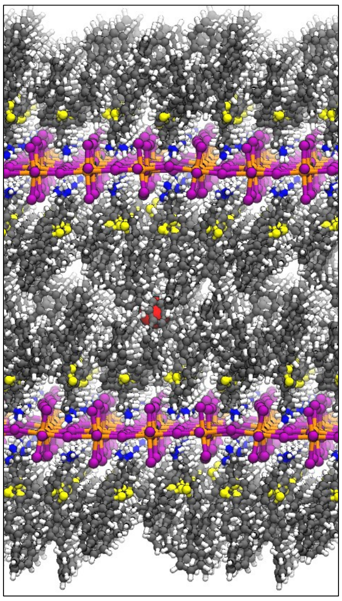

Supplementary Fig. 4 | Simulated stacked 2D perovskite for a typical structure of $(PPT)_2PbI_4$. The colors correspond as follows: purple, iodine atoms; orange, lead atoms; black, carbon atoms; grey, hydrogen atoms; blue, nitrogen atoms; yellow, sulfur atoms. The red sphere and its position denote the free anion I^- being pulled and the ending point of the ion diffusion, respectively. The stacked 2D perovskites with two repeating units were built, where the interface of the stacked structure defines the ending point of the ion diffusion.

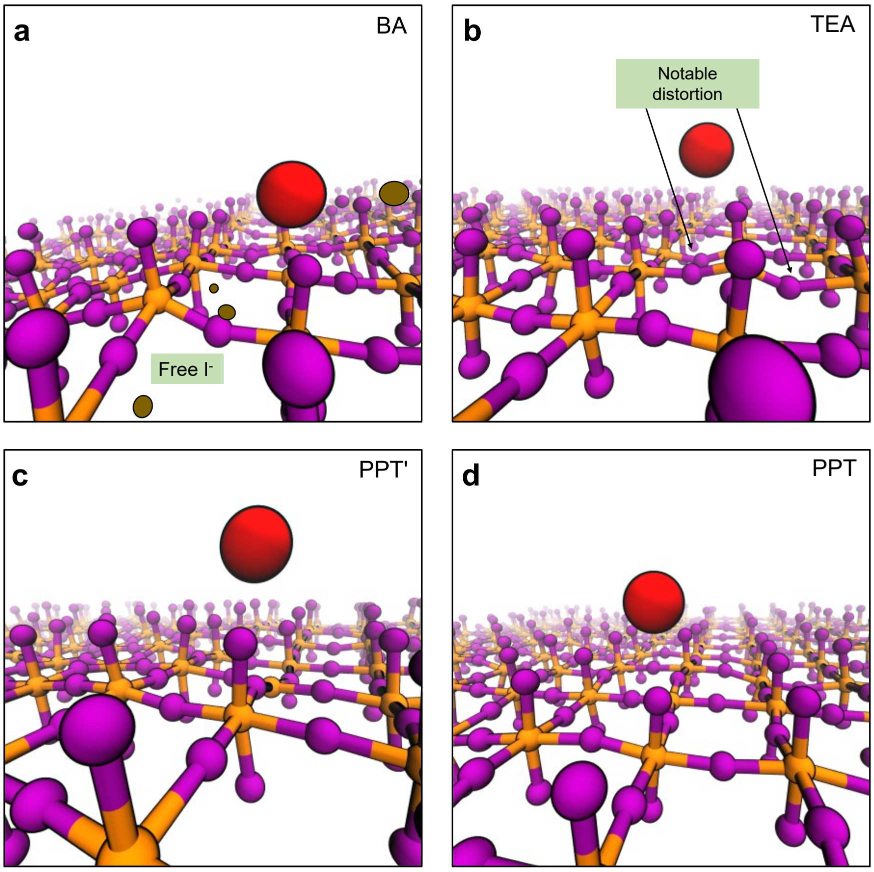

Supplementary Fig. 5 | Side view of snapshots from the MD simulations. a, $(BA)_2PbI_4$, **b,** $(TEA)_2PbI_4$, **c,** $(PPT')_2PbI_4$, **d,** $(PPT)_2PbI_4$. All organic ligands have been omitted from the snapshots for clarity. Orange, Pb atoms; purple, I atoms; red, magnified diffused I ions; brown, free I ions. The perspective is chosen so that the lattice is conspicuous and thus the size of the ion might differ with depth cueing on. The crystal structures of $(BA)_2PbI_4$ and $(TEA)_2PbI_4$ became distorted during the pulling processes (Supplementary Fig. 5a, b), so that several neighboring ions break free to diffuse (brown spheres in Supplementary Fig. 5a). In contrast, $(PPT')_2PbI_4$ and $(PPT)_2PbI_4$ can retain their crystal structures during ion diffusion (Supplementary Fig. 5c, d).

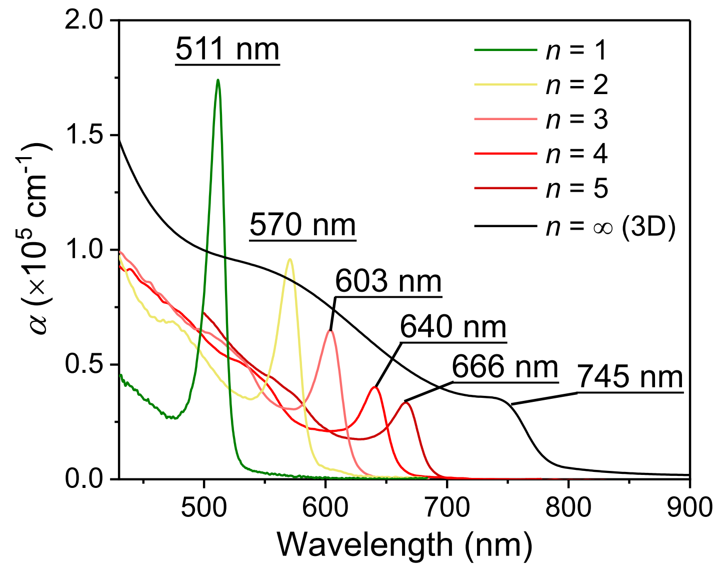

Supplementary Fig. 6 | Linear extinction coefficient α of lead iodide-based perovskites with n varying from 1 to 5, and to infinity (3D). Note, all the extinction coefficient data for n = 1 to 5 and 3D perovskites were taken from Refs. [2] and [3], respectively.

Based on the Lambert-Beer law, $A = \alpha \times b \times c$, where A is the absorbance, α is the absorption coefficient of specific n-phase, b is thickness of the film, c is the concentration of specific n-species, the absorbance should be the linear superposition of the absorbance of each n-phase. For a specific quasi-2D perovskite thin film, the thickness is the same. So, we can estimate the relative content of each n-phase in the film based on the absorption spectra and absorption coefficient. Supplementary Fig. 6 shows the absorption coefficient of n = 1, 2 ,3, 4, 5, and ∞ phase. The absorbance of specific film, for example PPT, could be expanded as, $A^{PPT} = \alpha_{n1} \times b \times c_{n1} + \alpha_{n2} \times b \times c_{n2} + \alpha_{n3} \times b \times c_{n3} + \alpha_{n4} \times b \times c_{n4} + \alpha_{n5} \times b \times c_{n5} + \ldots + \alpha_{n\infty} \times b \times c_{n\infty}$. The absorbance for BA, TEA, and PTT can be expanded in the same way. By doing linear superposition fitting, we can retrieve the pre-factor of absorption coefficient as ($b \times c_{n1}$). Then, the relative content of, for instance, n = 5 phase, can be determined as $(c\%)_{n5} = 100\% \times (b \times c_{n5})/[(b \times c_{n1}) + (b \times c_{n2}) + (b \times c_{n3}) + \ldots + (b \times c_{n\infty})]$.

This method can be generally used to estimate the n-phase distribution in all quasi-2D perovskites if the absorption coefficient for each n-phase is available. Unfortunately, the absorption coefficient data for high-n phase, such as n = 6, 7, …, are unavailable for the moment since it is quite difficult to grow such single crystal for the measurements. So, we could only roughly estimate the relative content by assuming that n = 6 phase has the same absorption coefficient as n = 5 phase and $n \geq 7$ phases have the same absorption coefficient as 3D phase. In this way, $A^{PPT} \approx \alpha_{n1} \times b \times$

c_{n1} + α_{n2} × b × c_{n2} + α_{n3} × b × c_{n3} + α_{n4} × b × c_{n4} + α_{n5} × b × c_{n5} + α_{n5} × b × c_{n6} + × $\alpha_{n\infty}$ × b × c_{n7} + + $\alpha_{n\infty}$ × b × $c_{n\infty}$. We can then solve a system of linear equations as follows:

A^{PPT} (511 nm) ≈ α_{n1}(511 nm) × b × c_{n1} + α_{n2}(511 nm) × b × c_{n2} + α_{n3}(511 nm) × b × c_{n3} + α_{n4}(511 nm) × b × c_{n4} + α_{n5}(511 nm) × b × c_{n5} +α_{n5}(511 nm) × b × c_{n6}+ × $\alpha_{n\infty}$(511 nm) × b × c_{n7} + +$\alpha_{n\infty}$(511 nm) × b × $c_{n\infty}$ (1)

A^{PPT} (570 nm) ≈ α_{n1}(570 nm) × b × c_{n1} + α_{n2}(570 nm) × b × c_{n2} + α_{n3}(570 nm) × b × c_{n3} + α_{n4}(570 nm) × b × c_{n4} + α_{n5}(570 nm) × b × c_{n5} +α_{n5}(570 nm) × b × c_{n6}+ × $\alpha_{n\infty}$(570 nm) × b × c_{n7} + +$\alpha_{n\infty}$(570 nm) × b × $c_{n\infty}$ (2)

A^{PPT} (603 nm) ≈ α_{n1}(603 nm) × b × c_{n1} + α_{n2}(603 nm) × b × c_{n2} + α_{n3}(603 nm) × b × c_{n3} + α_{n4}(603 nm) × b × c_{n4} + α_{n5}(603 nm) × b × c_{n5} +α_{n5}(603 nm) × b × c_{n6}+ × $\alpha_{n\infty}$(603 nm) × b × c_{n7} + +$\alpha_{n\infty}$(603 nm) × b × $c_{n\infty}$ (3)

A^{PPT} (640 nm) ≈ α_{n1}(640 nm) × b × c_{n1} + α_{n2}(640 nm) × b × c_{n2} + α_{n3}(640 nm) × b × c_{n3} + α_{n4}(640 nm) × b × c_{n4} + α_{n5}(640 nm) × b × c_{n5} +α_{n5}(640 nm) × b × c_{n6}+ × $\alpha_{n\infty}$(640 nm) × b × c_{n7} + +$\alpha_{n\infty}$(640 nm) × b × $c_{n\infty}$ (4)

A^{PPT} (666 nm) ≈ α_{n1}(666 nm) × b × c_{n1} + α_{n2}(666 nm) × b × c_{n2} + α_{n3}(666 nm) × b × c_{n3} + α_{n4}(666 nm) × b × c_{n4} + α_{n5}(666 nm) × b × c_{n5} +α_{n5}(666 nm) × b × c_{n6}+ × $\alpha_{n\infty}$(666 nm) × b × c_{n7} + +$\alpha_{n\infty}$(666 nm) × b × $c_{n\infty}$ (5)

A^{PPT} (680 nm) ≈ α_{n1}(680 nm) × b × c_{n1} + α_{n2}(680 nm) × b × c_{n2} + α_{n3}(680 nm) × b × c_{n3} + α_{n4}(680 nm) × b × c_{n4} + α_{n5}(680 nm) × b × c_{n5} +α_{n5}(680 nm) × b × c_{n6}+ × $\alpha_{n\infty}$(680 nm) × b × c_{n7} + +$\alpha_{n\infty}$(680 nm) × b × $c_{n\infty}$ (6)

A^{PPT} (700 nm) ≈ α_{n1}(700 nm) × b × c_{n1} + α_{n2}(700 nm) × b × c_{n2} + α_{n3}(700 nm) × b × c_{n3} + α_{n4}(700 nm) × b × c_{n4} + α_{n5}(700 nm) × b × c_{n5} +α_{n5}(700 nm) × b × c_{n6}+ × $\alpha_{n\infty}$(700 nm) × b × c_{n7} + +$\alpha_{n\infty}$(700 nm) × b × $c_{n\infty}$ (7)

A^{PPT} (745 nm) ≈ α_{n1}(745 nm) × b × c_{n1} + α_{n2}(745 nm) × b × c_{n2} + α_{n3}(745 nm) × b × c_{n3} + α_{n4}(745 nm) × b × c_{n4} + α_{n5}(745 nm) × b × c_{n5} +α_{n5}(745 nm) × b × c_{n6}+ × $\alpha_{n\infty}$(745 nm) × b × c_{n7} + +$\alpha_{n\infty}$(745 nm) × b × $c_{n\infty}$ (8)

The pre-factor of absorption coefficient, (b × c_n), can be then solved and, for instance, the relative content of n = 5-6 phases can be estimated based on $(c\%)_{n5\text{-}6}$ = 100% × (b × c_{n5} + b × c_{n6})/[(b × c_{n1}) + (b × c_{n2}) + (b × c_{n3}) + (b × c_{n4}) + (b × c_{n5}) + (b × c_{n6}) + (b × c_{n7}) + (b × $c_{n\infty}$)].

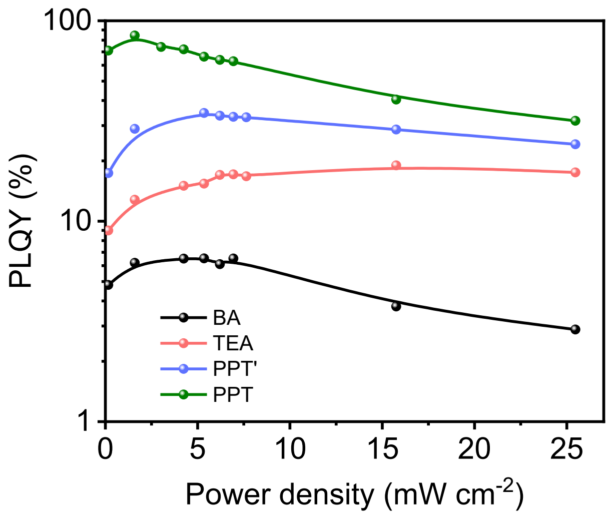

Supplementary Fig. 7 | Power dependent PLQY studies of quasi-2D perovskite thin films. The excitation source was a 375 nm continuous wave laser with tunable output power. To quantitatively compare their emission efficiencies, we examined the PLQYs for all the films with varied excitation powers. The PLQY gradually increased from 6.5% for BA, 18.9% for TEA, to 34.7% for PPT', and further to 84.4% for PPT. This high PLQY is indicative of a combination of less trap states, reduced exciton quenching effect, and enhanced energy transfer efficiency in the PPT film. Moreover, the PLQY of PPT film can be maintained at a high level of more than 71% at an excitation intensity as low as 0.2 mW cm^{-2}, confirming that the defect-mediated nonradiative recombination is insignificant[4].

Phase disproportionation necessarily involves the diffusion or transport of multiple different ions. More significant phase disproportionation indicates more severe ionic transport. During this transport, it will create a lot of vacancies and traps, such as in BA and TEA cases. In other words, less significant phase disproportionation will have subtle ionic transport, thus leading to less trap densities in the films, such as in PPT' and PPT cases.

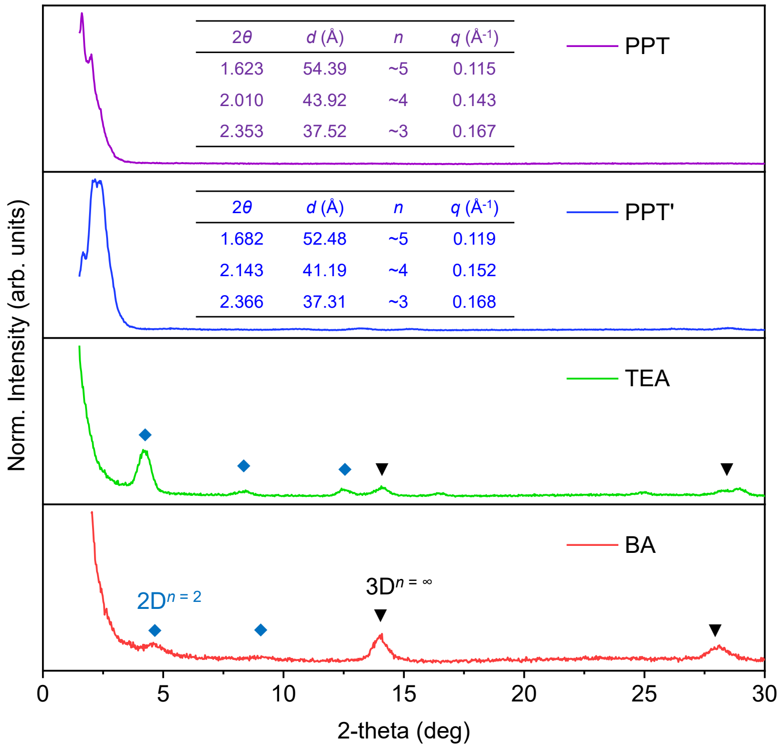

Supplementary Fig. 8 | XRD profiles of quasi-2D perovskite thin films. Both BA and TEA films exhibits obvious diffraction peaks corresponding to 2D (n = 2) phase and 3D ($n = \infty$) phase at the same time, which are indications of phase disproportion. For PPT'-based film, it doesn't show any diffraction peaks corresponding $n = \infty$ phase, suggesting the inhibition of 3D phase formation. Meanwhile, it shows three peaks at low angles as 2θ = 1.682°, 2.143°, and 2.366°, which are evidence of layered structures, and the interlayer spacings can be calculated as 52.48 Å, 41.19 Å, 37.31 Å, respectively. PPT film also does not show any 3D phase diffractions but with some low-angle diffraction peaks at 1.623°, 2.010°, 2.353°, which corresponds to an interlayer spacing of 54.39 Å, 43.92 Å, 37.52 Å. According to their single crystal structure (Supplementary Figs. 1, 2 and Table 1), we can roughly calculate the n number to be 5, 4, and 3, respectively, which indicates the only formation of median-n phases.

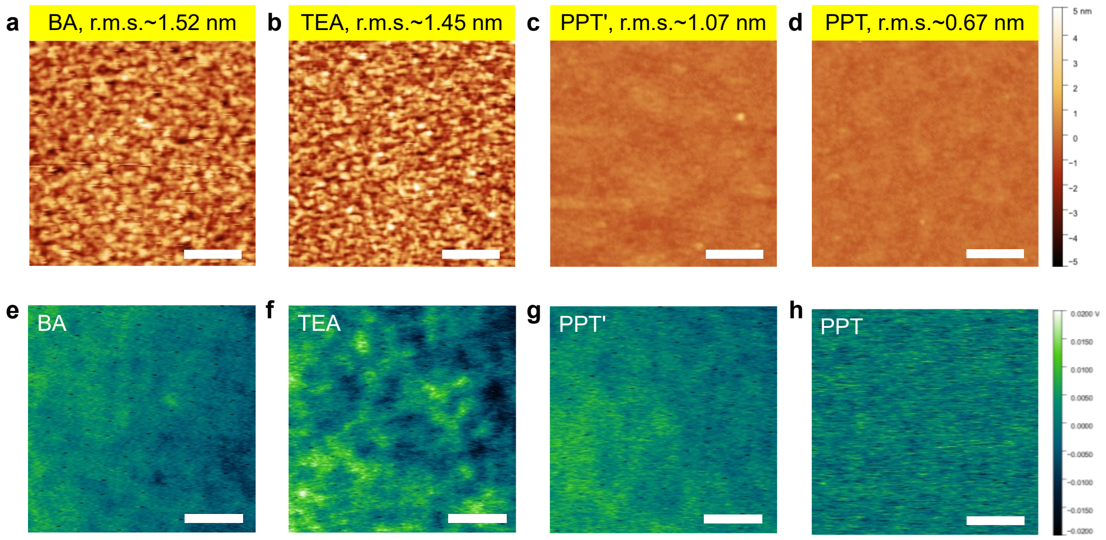

Supplementary Fig. 9 | The surface potential study. a-d, KPFM topography map and **e-h,** contact potential difference (CPD) map of quasi-2D perovskite thin films fabricated from different ligands. (a, e) BA, (b, f) TEA, (c, g) PPT', and (d, h) PPT. Scale bars, 1 μm. AFM height profile exhibits that the as-prepared PPT based thin film has a root-mean-square (r.m.s.) roughness of ~0.67 nm, which is lower than thin films prepared with other organic ligands (BA~1.52 nm, TEA~1.45 nm, PPT'~1.07 nm) and favorable for reducing current leakage in LEDs. Such a better surface morphology of PPT' and PPT samples could be understood as a result from a relatively slower crystal growth due to the much higher kinetic barrier of mass transport for crystallization provided by PPT' and PPT ligands. Regarding the slight roughness difference between PPT' and PPT films, the increased dihedral angle may induce better solubility for PPT ligand. Thereby, it will further slow-down the crystallization, leading to the formation of a more uniform film.

The surface potential is determined by KPFM relative to a Pt probe. All the KPFM images are processed with subtraction of the background. The surface potential of BA and TEA-based thin films exhibit substantial heterogeneity irrespective of grain boundaries and height profiles, which is correlated with inhomogeneous and broad phase distributions. The PPT' and PPT films possess more homogeneous surface potential. Among them, the CPD image of PPT film exhibits the most pronounced uniformity, which indicates a narrower and more homogenous *n*-phase distribution in the film. This is consistent with the UV-vis and PL studies.

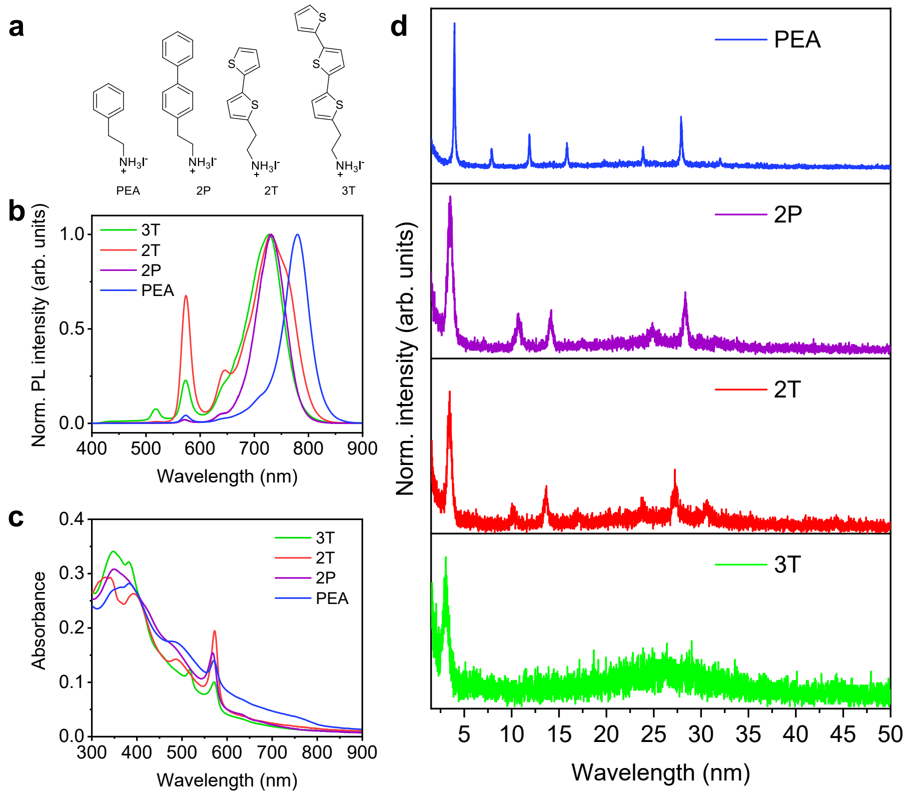

Supplementary Fig. 10 | Phase distribution studies with other ligands. a, Chemical structures of other organic ligands. **b,** PL; **c,** absorption; and **d,** XRD profiles of quasi-2D perovskite thin films fabricated from the ligands shown in (a).

All these ligands have been identified as conjugated ligands developed in our lab. After introducing them into the quasi-2D perovskites, we assessed their impact on phase distribution control by using PL, absorption, and XRD studies. The PEA ligand with shortest conjugation length leads to a thin film with a large amount of n = 2 and 3D phases, which can be evidenced from the PL emission at ~570 nm and ~780 nm, absorption at ~570 nm and ~760 nm. This broad phase distribution is an indication of phase disproportionation. By extending the conjugation length from PEA to 2P, we observed the suppression of 3D phase by noticing a shift of the main PL peak to ~730 nm with reduced absorbance at 3D phase. When replacing the phenyl rings in 2P with thiophenyl rings, the 2T ligand can also suppress the formation of 3D phase but favor the formation of n = 2 phase. By further extending the conjugation length to 3T, we achieved a thin film with increased relative contents of n = 1 and n = 2 phases free of $n = \infty$. These observations combined with the results and analysis in the main text clarify us that both the conjugation length and dihedral angle play a critical role in controlling the phase distribution in quasi-2D perovskites.

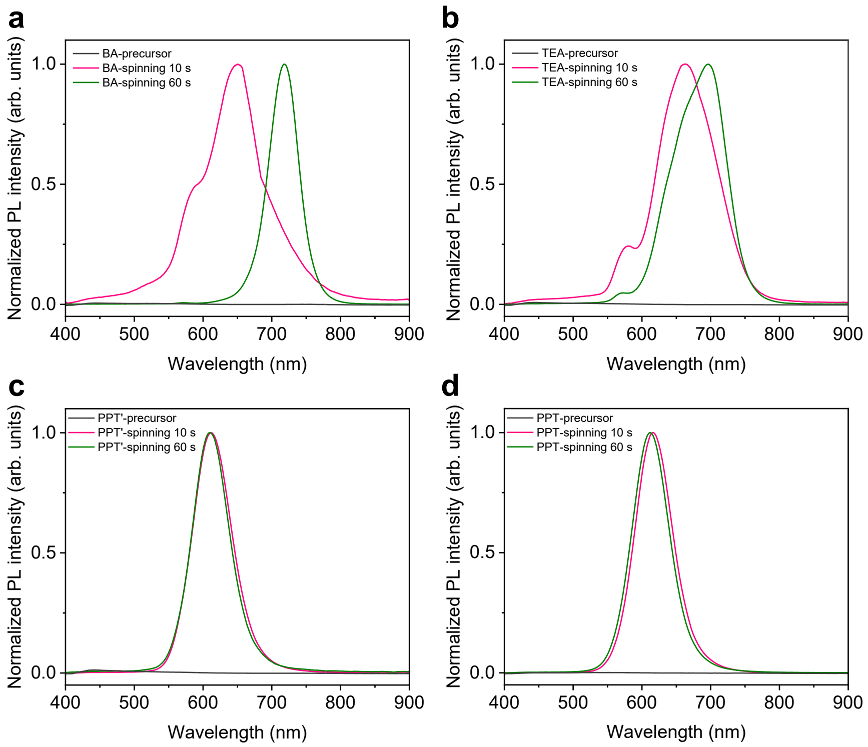

Supplementary Fig. 11 | Ex-situ PL studies of quasi-2D perovskite films during spin-coating and thermal annealing processes. a, BA; **b,** TEA; **c,** PPT'; **d,** PPT. At stage 1 (loading the perovskite precursors), all ligands-based films didn't exhibit noticeable PL emissions (black curves). This suggests the absence of any *n*-phase nucleation in this molecular state precursor solutions. After spinning (Stage 2), both BA- and TEA-based films exhibit broad PL emissions from median *n* (≈2-4) phases (pink curves in Supplementary Fig. 11a, b) for the first 10 seconds, implying the formation of kinetic products during their initial nucleation and growth. After continuously stirring for another 50 seconds, their PL emissions shift to higher *n*-phase (green curves in Supplementary Fig. 11a, b) controlled by thermodynamics, uncovering phase disproportionation in the spinning process. In sharp contrast, the PPT' and PPT films show pure PL emission from n = 3 phase during the whole spinning process (stage 2, pink and green curves in Supplementary Fig. 11c, d), which indicates the stabilization of kinetic intermediates during this initial crystallization process.

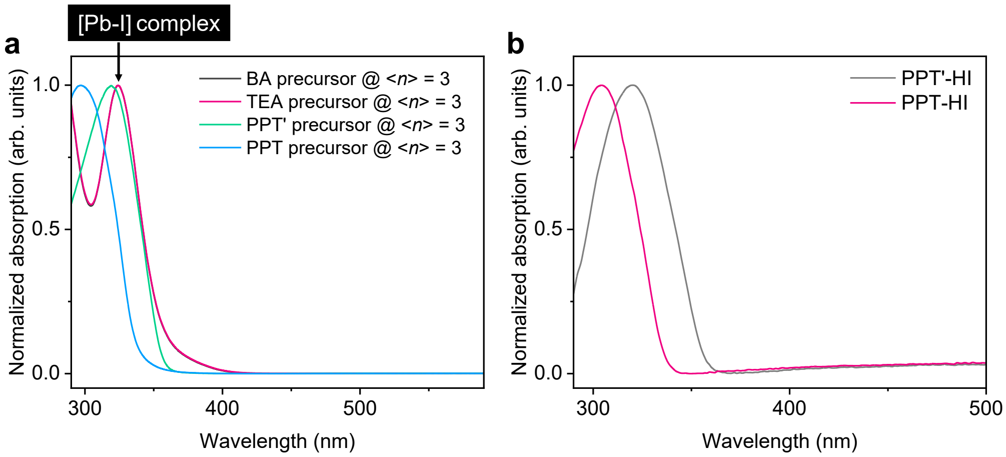

Supplementary Fig. 12 | Absorption spectra of DMF solutions. a, Perovskite precursor solutions in DMF; **b,** ligands solutions in DMF. The UV-vis spectra only show the absorption from [Pb-I] complex and large ligands themselves, suggesting that there is no specific *n*-phase nucleation in the solutions and these perovskite solutions could be considered as molecular precursor solution, i.e., homogeneous solutions of free molecules, cations, and anions.

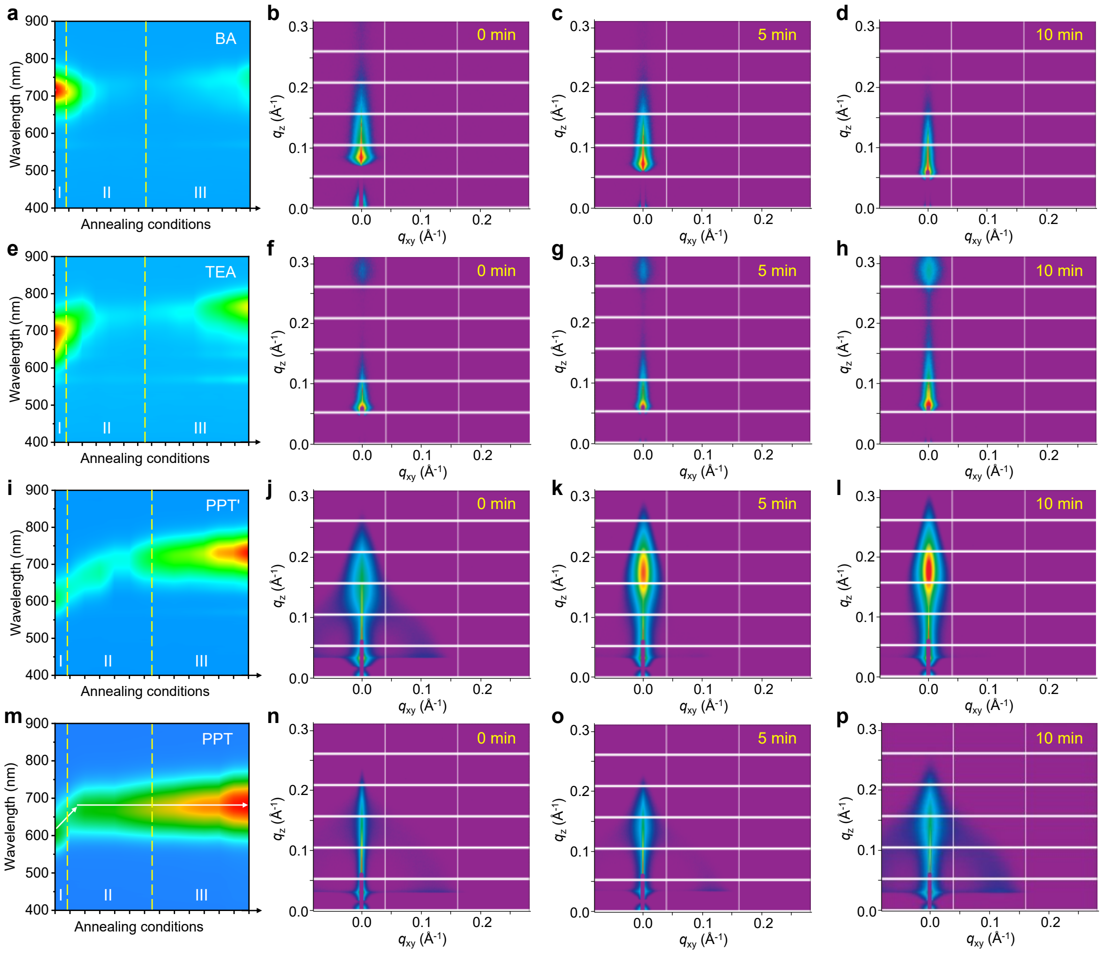

Supplementary Fig. 13 | Phase disproportionation study during the thermal annealing process. a-d, 2D plots of PL spectra for BA based film (a), and corresponding GISAXS patterns collected at different annealing times, 0 min (b), 5 min (c), 10 min (d). **e-h,** 2D plots of PL spectra for TEA based film (e), and corresponding GISAXS patterns collected at different annealing times, 0 min (f), 5 min (g), 10 min (h). **i-l,** 2D plots of PL spectra for PPT' based film (i), and corresponding GISAXS patterns collected at different annealing times, 0 min (j), 5 min (k), 10 min (l). **m-p,** 2D plots of PL spectra for PPT based film (m), and corresponding GISAXS patterns collected at different annealing times, 0 min (n), 5 min (o), 10 min (p). All the GISAXS patterns were collected at an incident angle of 0.2°.

Right after spin-coating (I), both BA- and TEA-based films exhibit PL emissions from n = 2 and $n \sim \infty$ phases (Supplementary Fig. 13a, e). During thermal annealing (II), their major PL emissions red shift towards 3D phase, which can be ascribed to a more thorough phase disproportionation. This phase evolution was also verified from the structural wise by corresponding GISAXS, where the diffraction patterns were

gradually shifted to the higher-n phases during thermal annealing (Supplementary Fig. 13b-d, f-h). In contrast, both PPT' and PPT film show pure PL emission (Supplementary Fig. 13i, m) from $n = 3$ phase before thermal annealing (I), indicating the stabilization of kinetic intermediates during the initial crystallization stage. After thermal annealing, the PPT' film exhibits moderate disproportionation into $n = 2$ and $n \geq 8$ phases (II and III in Supplementary Fig. 13i). Interestingly, the PPT film shows steady PL emission with a narrower phase distribution of n~2-6 after thermal annealing (II and III in Supplementary Fig. 13m), suggesting the occurrence of slight phase disproportionation. The evolution of GISAXS pattern for both PPT' and PPT ligands only shows a marginal expansion during different annealing time, verifying the suppression of phase transformation by our developed conjugated ligands, comparing to the BA and TEA cases.

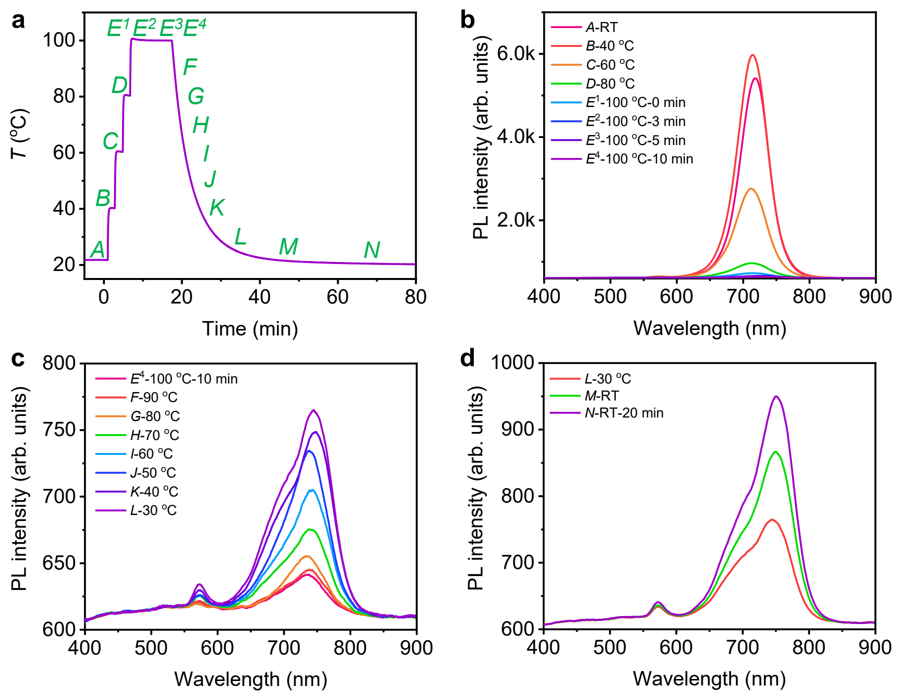

Supplementary Fig. 14 | In-situ PL study of BA-based quasi-2D perovskite thin film. a, The heating procedures used in this in-situ study. Each letter labels the time point where we took the PL spectra, as plotted in (b-d). **b,** PL spectra taken from the thermal annealing stage. **c,** PL spectra taken from the cooling stage. **d,** PL spectra taken after a long time relax at room temperature. These curves are the original data for the 2D plot of PL spectra in Supplementary Fig. 13a. Right after spin-coating, BA film exhibits broad n distribution with obvious PL emissions from $n = 2$ and $n \sim \infty$ phases. During thermal annealing, the PL emissions red shift towards 3D phase accompanied with enhanced PL emission from $n = 2$ and 3 phases, which is an indication of accelerated phase disproportionation. After cooling down, the PL emission is enhanced without obvious shift, which originated from reduced thermal quenching in perovskite thin films.

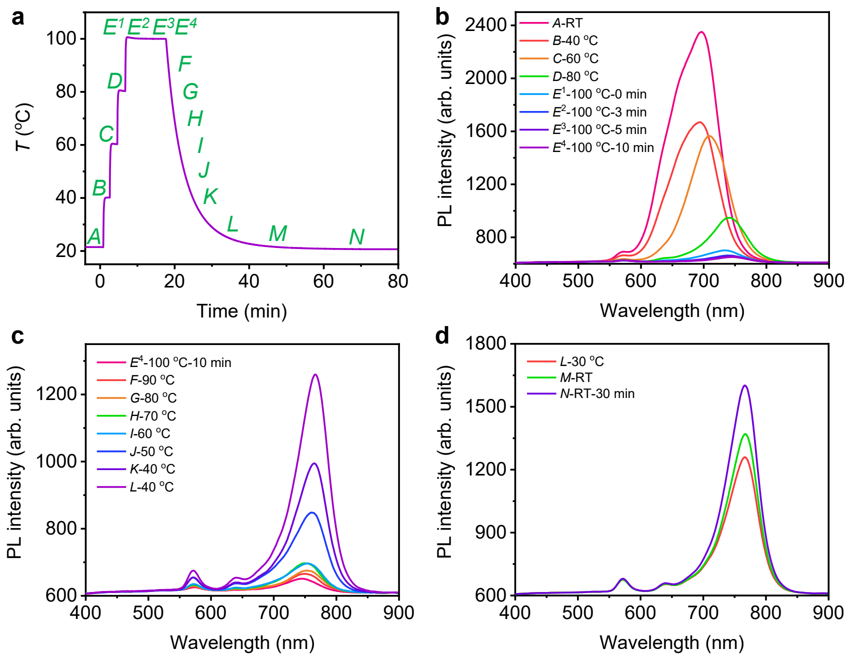

Supplementary Fig. 15 | In-situ PL study of TEA-based quasi-2D perovskite thin film. a, The heating procedures. Each letter labels the time point where we took the PL spectra, as plotted in (b-d). **b,** PL spectra taken from the thermal annealing stage. **c,** PL spectra taken from the cooling stage. **d,** PL spectra taken after a long time relax at room temperature. These curves are the original data for the 2D plot of PL spectra in Supplementary Fig. 13e. Similar to the BA film, TEA film experiences obvious phase disproportionation right after spin-coating. After thermal annealing, the accelerated phase disproportionation eventually leads to a red shift of PL emission towards 3D phase accompanied with enhanced PL emission from n = 2 and 3 phases. The PL emission enhancement after cooling down could be attributed to reduced thermal quenching effect.

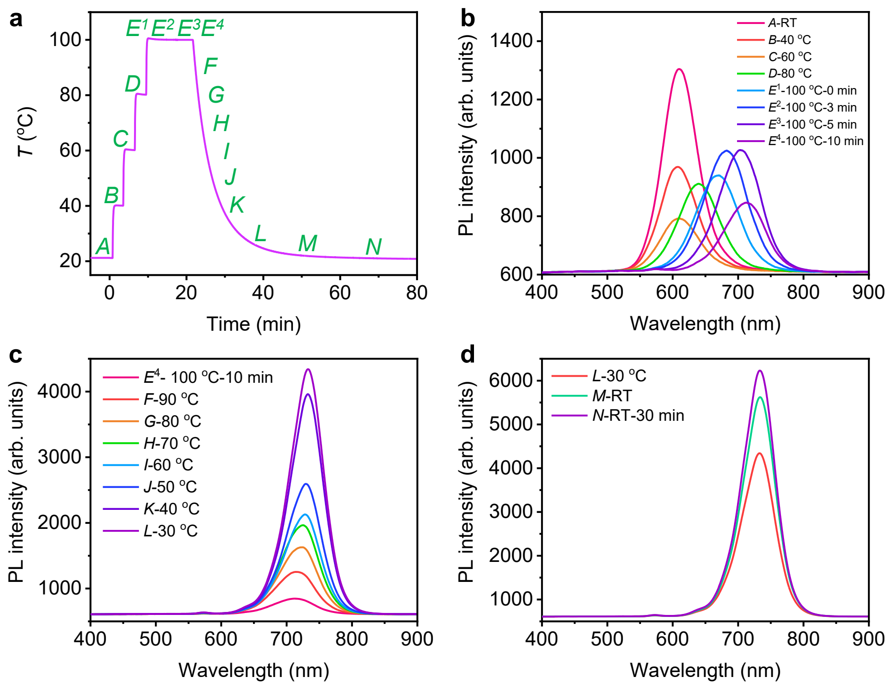

Supplementary Fig. 16 | In-situ PL study of PPT'-based quasi-2D perovskite thin film. a, The heating procedures. Each letter labels the time point where we took the PL spectra, as plotted in (b-d). **b,** PL spectra taken from the thermal annealing stage. **c,** PL spectra taken from the cooling stage. **d,** PL spectra taken after a long time relax at room temperature. These curves are the original data for the 2D plot of PL spectra in Supplementary Fig. 13i. For the PPT' film, it shows pure PL emission from n = 3 phase before thermal annealing, which indicates the formation of stabilized kinetic intermediates free of phase disproportionation during the initial crystallization. However, after thermal annealing, PPT' film proceeded with disproportionation into n = 2 and $n \geq 8$ phases, which essentially suggests that thermal energy helps overcome the kinetic barrier for the disproportionation reaction in PPT' based perovskite solids. The significant PL emission enhancement after cooling down was also ascribed to reduced thermal quenching effect.

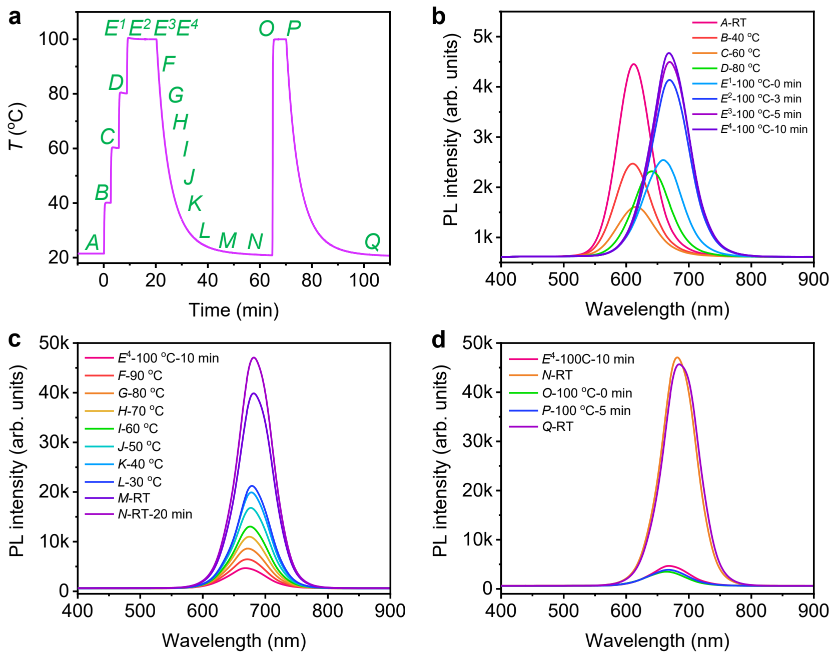

Supplementary Fig. 17 | In-situ PL study of PPT-based quasi-2D perovskite thin film. a, The heating procedures. Each letter labels the time point where we took the PL spectra, as plotted in (b-d). **b,** PL spectra taken from the thermal annealing stage. **c,** PL spectra taken from the cooling stage. **d,** PL spectra taken after a long time relax at room temperature. These curves are the original data for the 2D plot of PL spectra in Supplementary Fig. 13m. More significantly, the PPT ligand can effectively stabilize the kinetic products and suppress phase disproportionation during both the initial crystallization and subsequent crystal growth, as evidenced by the well-defined PL emission from median *n*-phases before and after heating. Notably, the PL emission still red shifts during thermal annealing process, suggesting the occurrence of slight phase disproportionation. But this red shift is less significant compared to the BA, TEA, and PPT' cases, indicating a better suppression of phase disproportionation by PPT ligand. This thus leads to a high-quality quasi-2D perovskite film with controlled phase distribution.

In addition, we heated up the film again to 100 °C for 5 minutes, which results in a decreased PL emission (green and blue curves in Supplementary Fig. 17d) with similar intensity to that during the first-round annealing (purple curve in Supplementary Fig. 17b). After cooling down to room temperature, the PL intensity ramp back to its initial level (purple and orange curves in in Supplementary Fig. 17d), which proves the reduced thermal quenching effect as the major reason for the significant PL enhancement after cooling down.

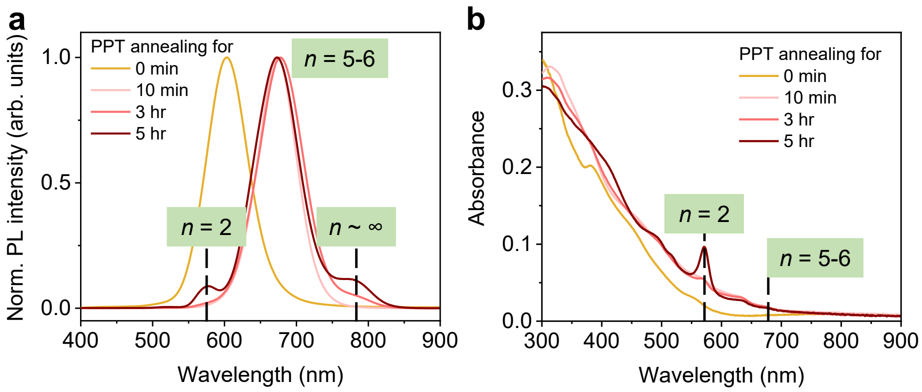

Supplementary Fig. 18 | Spectroscopic study of PPT-based quasi-2D perovskite thin film during a long-term thermal annealing. a, PL; **b,** absorption spectra. Upon heating for a long period (e.g., 100 ºC for 5 hours), the PL spectra of PPT film shows emerging shoulder peaks at 576 and 784 nm, which correspond to n = 2 and $n \sim \infty$ phases, respectively. In addition, the absorption at 674 nm (corresponding to n = 5 phase) decreases accompanied with a significantly enhanced absorption peak at 572 nm (corresponding to n = 2 phase). These further demonstrate that median n-phases are kinetic products and they eventually experienced phase disproportionation into lower- and higher-n phases simultaneously if enough thermal energy or time is provided.

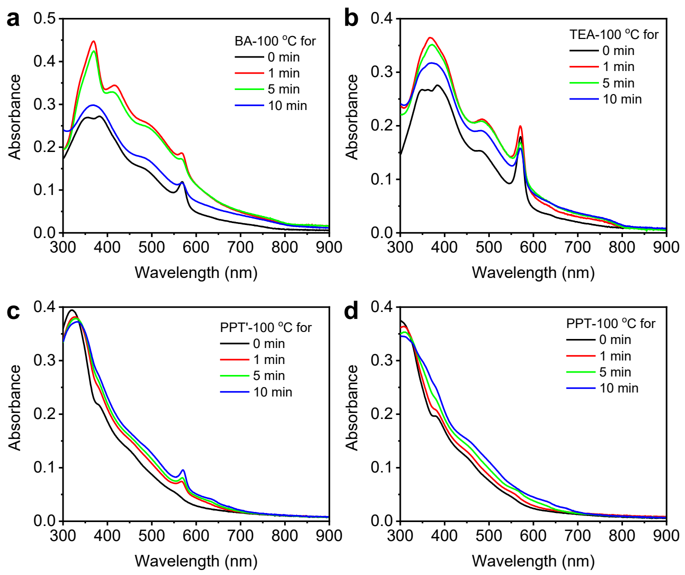

Supplementary Fig. 19 | Ex-situ absorption spectra of quasi-2D perovskite thin films at different annealing stages. a, BA; **b,** TEA; **c,** PPT'; **d,** PPT. Before thermal annealing, both BA and TEA-based films exhibit broad n distribution with obvious absorption peaks from n = 2 and $n \sim \infty$ phases, suggest the occurrence of phase disproportionation at the initial crystallization. During thermal annealing, their absorbance at n = 2 and $n \sim \infty$ are both enhanced. For the PPT' film, its absorption is dominated by n = 2-3 phases before thermal annealing, which is an indication of stabilizing kinetic intermediates and suppressing phase disproportionation during the initial crystallization. After thermal annealing, PPT' film disproportions into n = 2 and $n \geq 8$ phases (blue curve in Supplementary Fig. 19c), suggesting that the thermal energy helps overcome the energy barrier of this disproportionation reaction in PPT' based perovskite solids. With further developing in ligands, PPT ligands can effectively stabilize the kinetic products and suppress phase disproportionation during both the initial crystallization and crystal growth, thus leading to a narrower phase distribution as evidenced by the absorption peaks from median n-phases (Supplementary Fig. 19d).

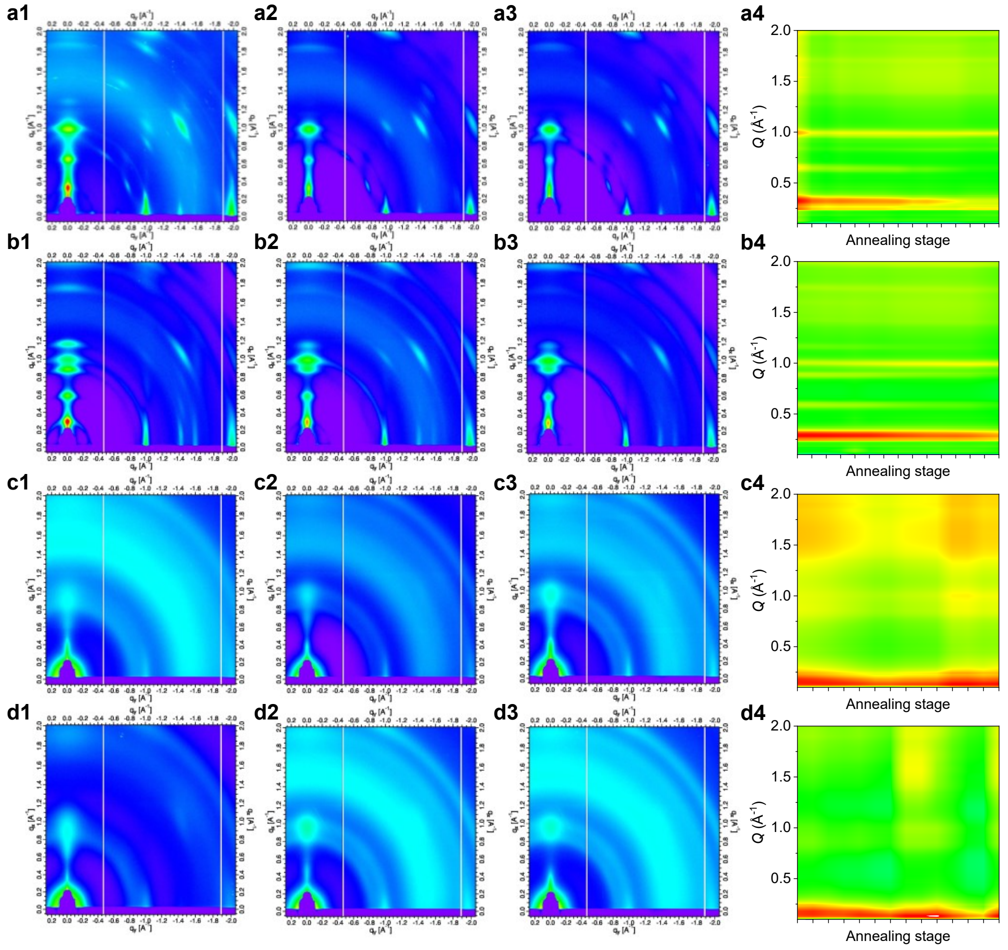

Supplementary Fig. 20 | In-situ GIWAXS of quasi-2D perovskite thin film annealed at 100 °C. a1-a3, BA-based quasi-2D perovskite thin film before annealing (a1), annealed for 10 min (a2), and after cooling down to room temperature (a3); **a4,** Time-dependent evolution of *q* diffractions. **b1-b3,** TEA-based quasi-2D perovskite thin film before annealing (b1), annealed for 10 min (b2), and after cooling down to room temperature (b3); **b4,** Time-dependent evolution of *q* diffractions. **c1-c3,** PPT'-based quasi-2D perovskite thin film before annealing (c1), annealed for 10 min (c2), and after cooling down to room temperature (c3); **c4,** Time-dependent evolution of *q* diffractions. **d1-d3,** PPT-based quasi-2D perovskite thin film before annealing (d1), annealed for 10 min (d2), and after cooling down to room temperature (d3); **d4,** Time-dependent evolution of *q* diffractions.

At I (right after spin-coating), both BA- and TEA-based films exhibit broad n distribution with obvious diffraction from $n \sim \infty$ ($q_z \sim 1.0$ Å^{-1}), median n ($q_z < 0.5$ Å^{-1}), and $n = 2$ ($q_z \sim 0.5$-0.75 Å^{-1}) phases (Supplementary Fig. 20a1, b1), which indicate the occurrence of phase disproportionation during the initial crystallization. After thermal annealing (II and III), it is clear to see that the peaks corresponding to 3D

phase remain strong and peaks corresponding to median-*n* phases become weaker (Supplementary Fig. 20a2-a4, b2-b4), suggesting that thermal energy further accelerated phase disproportionation in BA and TEA based films. For PPT' based quasi-2D perovskites, there is negligible 3D phase formation before annealing compared with BA and TEA cases (Supplementary Fig. 20c1), suggesting the suppression of phase disproportionation during the initial crystallization in PPT' film. Nevertheless, after thermal annealing, the PPT' film shows enhanced diffraction from 3D phase (Supplementary Fig. 20c2-c4), which is an indication of phase disproportionation. In sharp contrast, PPT based quasi-2D perovskites exhibit negligible 3D phase formation during both the spin-coating and thermal annealing stages, and the median-*n* phases remain intense even after annealing (Supplementary Fig. 20d1-d4), demonstrating the better suppression of phase disproportionation by PPT ligand.

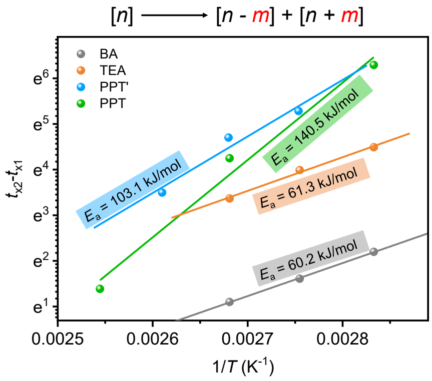

Supplementary Fig. 21 | Extracted average activation energy of disproportion reaction based on isothermal annealing kinetic studies. The phase disproportionation reaction can be simplified as a phase transformation from middle [*n*] phase to a lower [*n* - *m*] phase and a higher [*n* + *m*] phase. Referring to the kinetic model for solid state transformation, we use the following equation (1)[5,6] to retrieve the average activation energy for our solid-state phase disproportionation:

$$\ln (t_{x2}-t_{x1}) = E_a/(RT) - \ln k_0 + \ln (\beta_{x2} - \beta_{x1}) \quad (1)$$

Where t_{xn} is the time at which the transformed fraction is x_n, E_a is the average activation energy, R is the ideal gas constant, T is the temperature, k_0 is the rate constant pre-factor, and β_{xn} is a state property that is invariant to the time/temperature path. The introduction of β_{xn} assumes that there is some state property that is monotonically related to the physical property being measured. To apply equation, data is plotted as $\ln (t_{x2} - t_{x1})$ versus $1/T$ based on Supplementary Figs. 22-25 and the slope of the fitted line can be used to calculate E_a after multiplied by R. In our case, t_{x1} and t_{x2} represent the time at which the relative absorbance is 1.0 and 0.9, respectively. The slopes of the fitted kinetic curves for BA, TEA, PPT', and PPT are 7.24×10^3, 7.37×10^3, 1.24×10^4, 1.69×10^4 K^{-1}, respectively. Accordingly, the average E_a for phase disproportionation is estimated to be 60.2, 61.3, 103.1, 140.5 kJ mol^{-1} for BA, TEA, PPT', and PPT film, respectively. This E_a trend is consistent with anion diffusion study on the $n = 1$ systems shown in Fig. 1n in the main text. It should be noted that the extracted E_a is an average activation energy for taking the phase disproportionation as a net reaction, where the activation energies for different pathways (e.g., disproportionation into different lower-*n* or higher-*n* phase) are still coupled. Although the present data is insufficient to determine specific pathways, an average E_a can still provide key insight into the crystallization process and the extent to which it can be affected by changes to the reaction mechanisms. Particularly, the average activation energy estimated from PPT is slightly higher than that of PPT', which could be ascribed to the increased dihedral angle and reduced planarity.

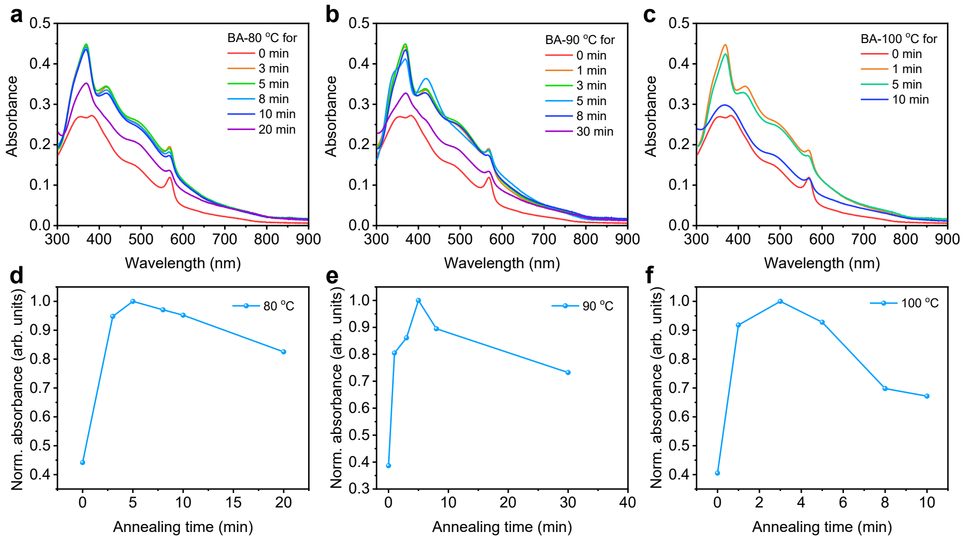

Supplementary Fig. 22 | Kinetic data for isothermally annealed BA samples. a-c, The evolution of absorption spectra at different annealing times; **d-f,** the extracted absorbance around 635 nm for annealing at (a, d) 80 °C; (b, e) 90 °C; (c, f) 100 °C. The absorbance at 635 nm corresponds to median *n* phases, which was chosen for the following kinetic analysis. The maximum absorbance at each annealing temperature was normalized to 1.0. These data were plotted as ln (t_{x2} − t_{x1}) versus 1/T in Supplementary Fig. 21 and the slope of the fitted line can be used to calculate E_a.

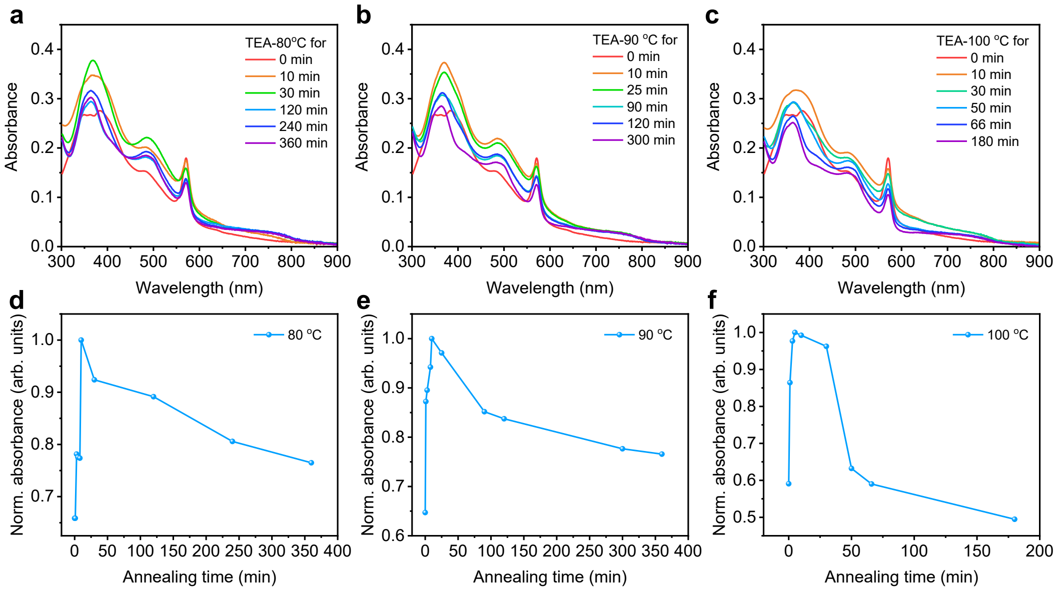

Supplementary Fig. 23 | Kinetic data for isothermally annealed TEA samples. a-c, The evolution of absorption spectra at different annealing times; **d-f,** the extracted absorbance around 635 nm for annealing at (a, d) 80 °C; (b, e) 90 °C; (c, f) 100 °C. The absorbance at 635 nm corresponds to middle *n* phases, which was chosen for the following kinetic analysis. The maximum absorbance at each annealing temperature was normalized to 1.0. These data were plotted as ln (t_{x2} − t_{x1}) versus $1/T$ in Supplementary Fig. 21 and the slope of the fitted line can be used to calculate E_a.

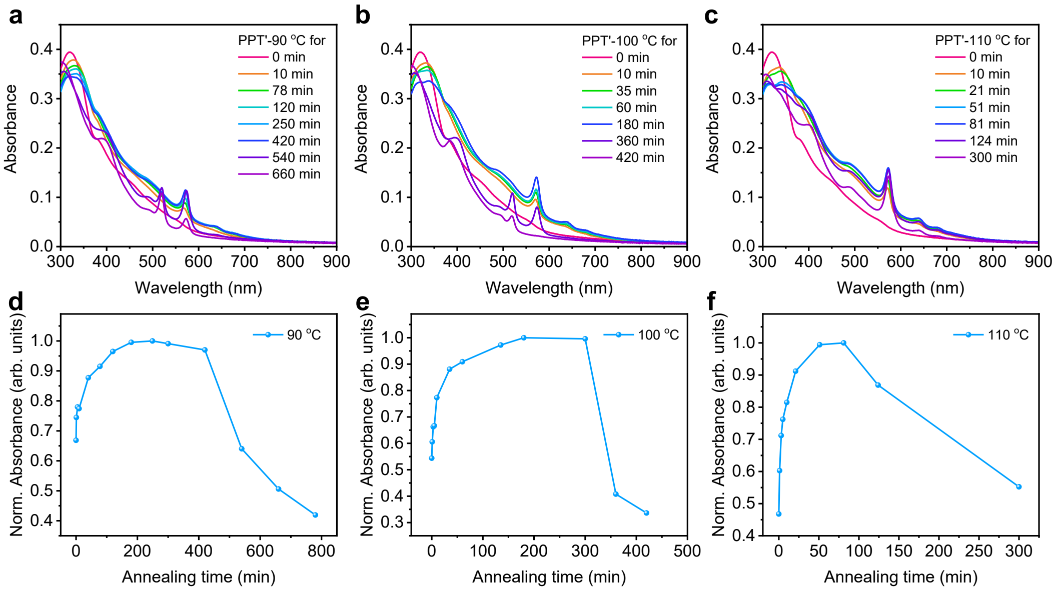

Supplementary Fig. 24 | Kinetic data for isothermally annealed PPT' samples. a-c, The evolution of absorption spectra at different annealing times; **d-f,** the extracted absorbance around 680 nm for annealing at (a, d) 90 °C; (b, e) 100 °C; (c, f) 110 °C. The absorbance at 680 nm corresponds to middle *n* phases, which was chosen for the following kinetic analysis. The maximum absorbance at each annealing temperature was normalized to 1.0. These data were plotted as ln (t_{x2} − t_{x1}) versus 1/T in Supplementary Fig. 21 and the slope of the fitted line can be used to calculate E_a.

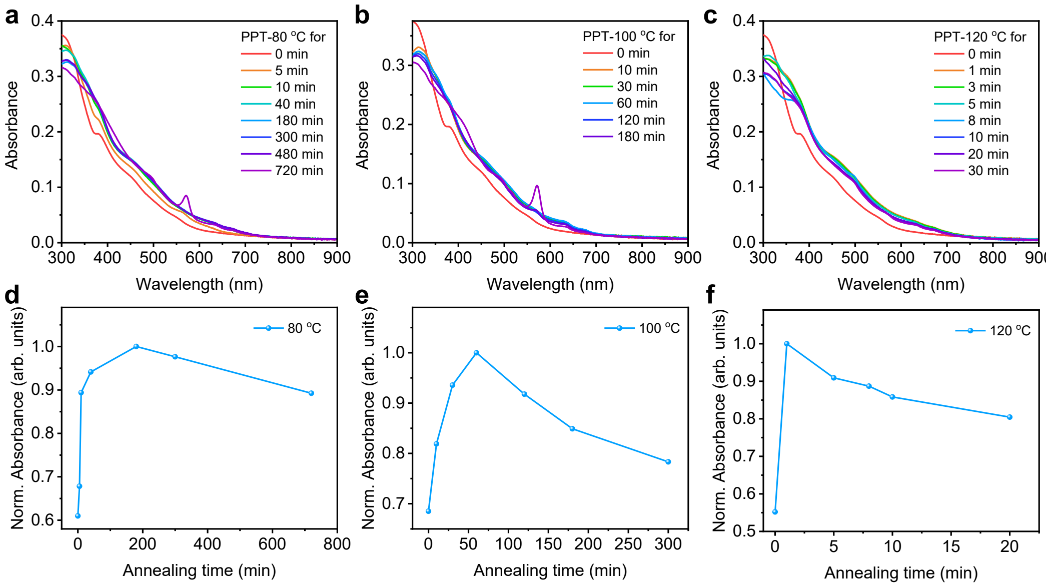

Supplementary Fig. 25 | Kinetic data for isothermally annealed PPT samples. a-c, The evolution of absorption spectra at different annealing times; **d-f,** the extracted absorbance around 680 nm for annealing at (a, d) 80 °C; (b, e) 100 °C; (c, f) 120 °C. The absorbance at 680 nm corresponds to middle *n* phases, which was chosen for the following kinetic analysis. The maximum absorbance at each annealing temperature was normalized to 1.0. These data were plotted as ln (t_{x2} − t_{x1}) versus 1/T in Supplementary Fig. 21 and the slope of the fitted line can be used to calculate E_a.

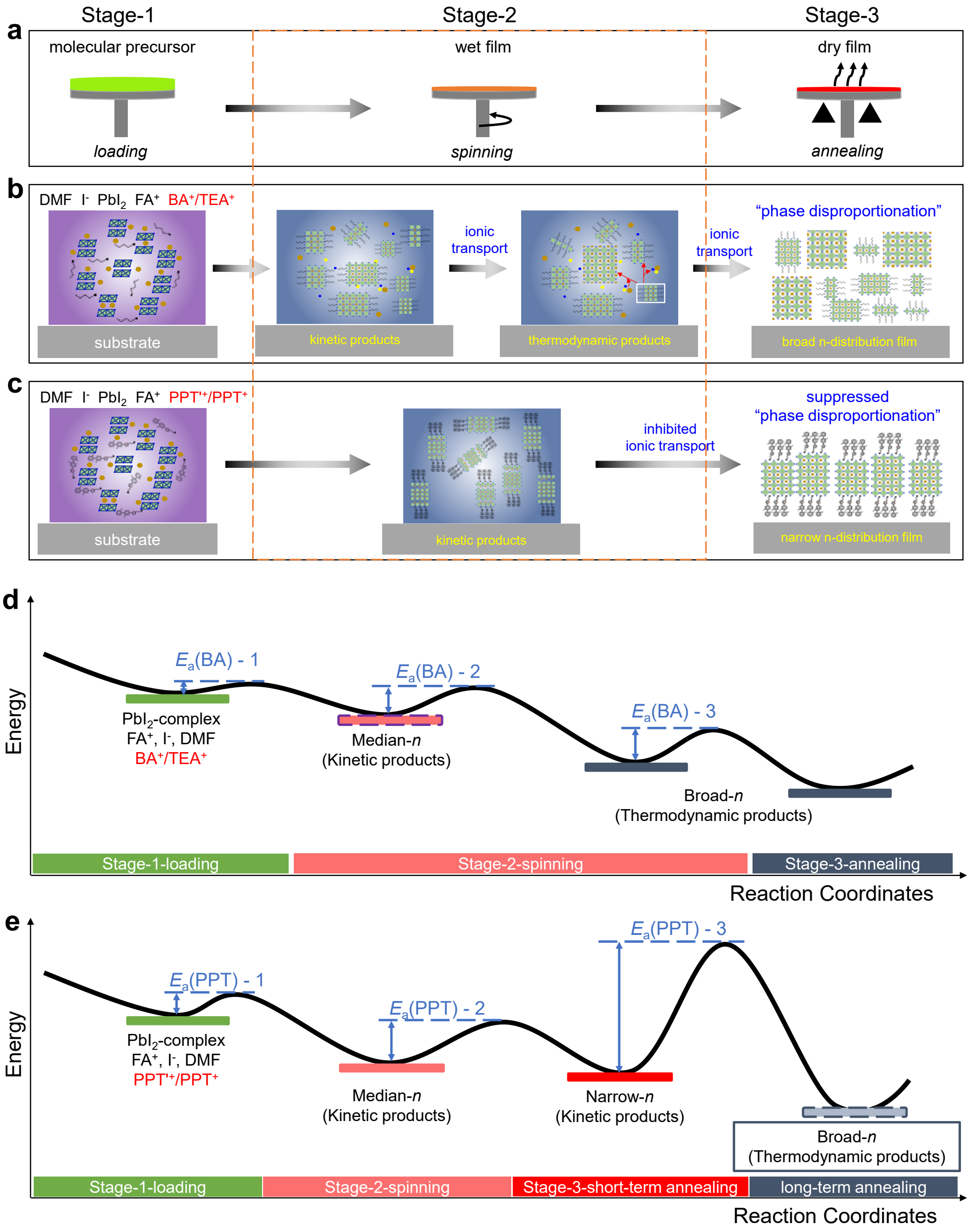

Supplementary Fig. 26 | Schematic diagram of proposed ligand-mediated crystallization mechanism. a, Illustration for the spin coating and thermal annealing processes. Stage-1, loading the perovskite molecular precursor solution; Stage-2, spin-coating the precursor solution; Stage-3, thermal annealing the spin-coated wet film. **b,** BA and TEA; **c,** PPT' and PPT based crystallization schemes obtained from the combination of ex-situ PL (Supplementary Fig. 11), in-situ PL (Supplementary Fig. 13) and corresponding GISAXS (Supplementary Fig. 13) characterizations. Proposed energy diagram of phase transformation. **d,** BA and TEA; **e,** PPT' and PPT.

The perovskite molecular precursors will nucleate as kinetic intermediates first no matter what kinds of ligands are used (pink curves in Supplementary Fig. 11),

followed by disproportionation into lower and higher-n phases if enough energy or time is supplied to overcome the kinetic barrier of mass transport. Specifically, for BA and TEA, kinetically trapped median-n phases (n = 2~4) can only be observed at the very beginning of spin-coating and they quickly evolve to higher-n phases, which is determined by thermodynamics (pink and green curves in Supplementary Fig. 11, middle panel in Supplementary Fig. 26b). After thermal annealing, significant phase disproportionation happens, thus leading to broad n-distribution (right panel in Supplementary Fig. 26b). In contrast, both PPT' and PPT can retain n = 3 kinetic intermediate phases very well during the whole spinning process (pink and green curves in Supplementary Fig. 11, middle panel in Supplementary Fig. 26c). And they slightly disproportionated into a narrow n-phase distribution after thermal annealing due to the diffusion-limited phase transformation in the solid state (right panel in Supplementary Fig. 26c).

By using different ligands, they might share similar initial nucleation pathways (diffusion-less) to form kinetic products since the kinetic products usually have a lower energy barrier than that of thermodynamic products. However, their crystal growth pathways are significantly different, where BA and TEA show very fast crystal growth into broad phase distribution, while PPT' and PPT are much slower with a narrower phase distribution. This ligand-dependent crystallization behavior regarding different growth rates can be also attributed to the different kinetic barriers provided by ligands during the phase transformation. Moreover, the activation energy labelled as E_a(BA)-3 (Supplementary Fig. 26d) and E_a(PPT)-3 (Supplementary Fig. 26e) could be understood as the average energy determined from the phase disproportionation kinetics studies shown in Supplementary Fig. 21.

We noticed that by heating the PPT film at 100 °C for long periods (> 5 hours), small amounts of n = 2 and $n \sim \infty$ phases appeared (Supplementary Fig. 18). This further demonstrates the median-n phases are the kinetic products and they eventually undergo phase disproportionation, leading to a broad-n distribution of thermodynamic products for PPT case (long-term annealing stage in Supplementary Fig. 26e). In short, our newly designed conjugated bulky ligands, PPT' and PPT, are good at suppressing this ion diffusion-limited phase disproportionation, leading to a relatively narrow n-phase distribution. PPT is slightly better than PPT' in terms of phase control behaviors since the increased dihedral angle by two methyl groups at 2- and 2'-positions would result in better solubility. This is expected to enhance the solution processability and film morphology, thus leading to better control over phase distribution.

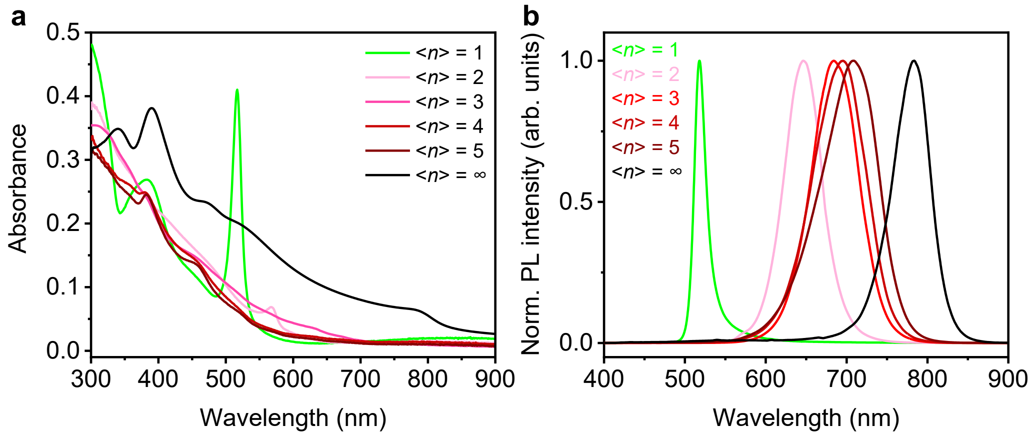

Supplementary Fig. 27 | Spectroscopic study of PPT-based quasi-2D perovskite films fabricated from precursor solutions with different *n* values. a, Absorption; **b,** PL spectra. For instance, the $<n> = 3$ represents that the stoichiometry ratio of precursor solution follows the general chemical formula of $(PPT)_2FA_{n-1}Pb_nI_{3n+1}$ with a nominal $n = 3$. The emission wavelengths can be rationally tuned from 650 to 710 nm by adjusting the stoichiometry ratio of precursor solutions from $<n> = 2$ to $<n> = 5$, which confirms a tunable phase distribution via a better control over the phase disproportionation.

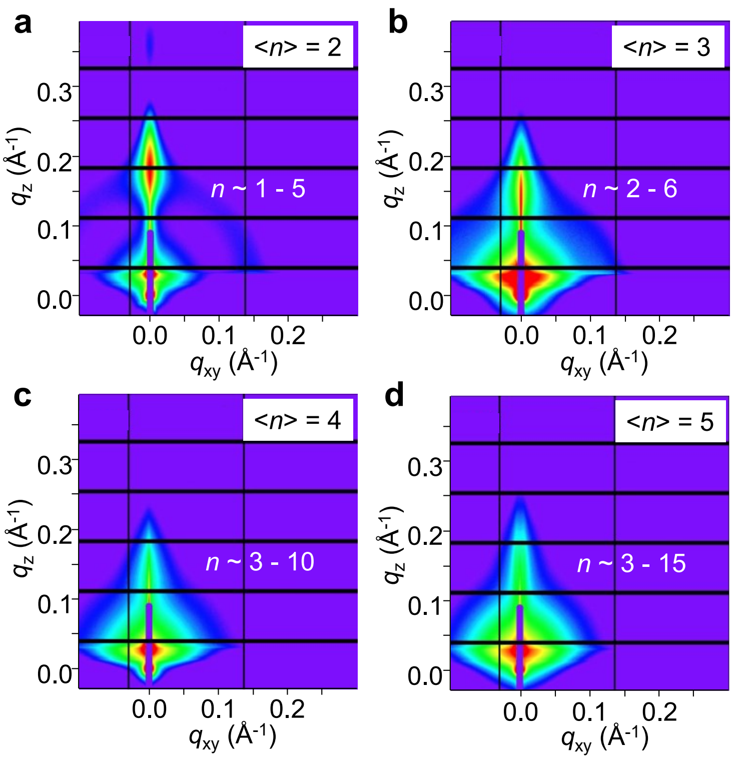

Supplementary Fig. 28 | GISAXS patterns of PPT based quasi-2D perovskite films fabricated from precursors with varied *n* values. a, <*n*> = 2; **b,** <*n*> = 3; **c,** <*n*> = 4; **d,** <*n*> = 5. We can estimate the exact *n* phase distribution in the films to be ~1-5, ~2-6, ~3-10, ~3-15 for nominal <*n*> = 2, 3, 4, 5 precursor solutions, respectively. These results further validate that PPT ligand can provide good control over phase distribution.

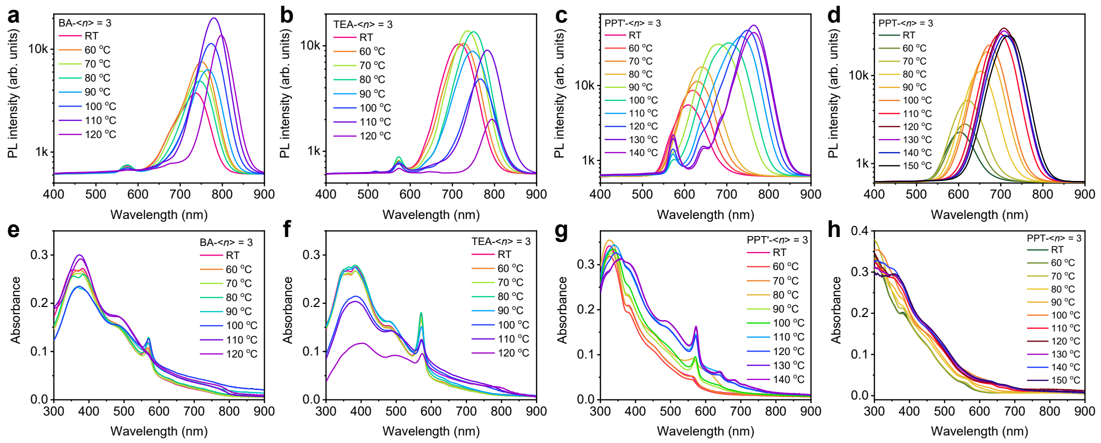

Supplementary Fig. 29 | Annealing temperature dependent PL (top) and absorption (bottom) spectra of quasi-2D perovskite. a,e, BA; **b,f,** TEA; **c,g,** PPT'; **d,h**, PPT. Note: the annealing time for all films is set to 10 min.

For BA and TEA films, we clearly observed the red shift of PL emissions by increasing the annealing temperature (Supplementary Fig. 29a, b), indicating accelerated phase disproportionation with elevated temperatures. In terms of PPT' films, we also observed such a red shift in the PL spectra with increasing the annealing temperature, but less significant compared with the BA and TEA cases. This suggests that PPT' can suppress the phase disproportionation and the kinetic barrier provided by PPT' ligand is higher than that of BA and TEA. Above 100 °C, PPT' film shows more broad emissions with the obvious emission from n = 2 (Supplementary Fig. 29c), further suggesting that a substantial phase disproportionation occurs in PPT' film and it was thermally activated. In other word, the kinetic barrier provided by PPT' ligand would be overcome by annealing at 100 °C for 10 min with noticeable rate of phase disproportionation. Interestingly, the PPT film only shows gradually red shift in the PL spectra without obvious shoulder peaks from other n-phases up to 150 °C (Supplementary Fig. 29d). This should be ascribed to the suppression of phase disproportionation by PPT with elevated kinetic barrier. The red shift in PPT film is also an indication of phase disproportionation, but less significant compared to the other cases. All the absorption spectra in Supplementary Fig. 29e-h agree well with our above analyses.

Overall, the phase disproportionation in quasi-2D perovskite films could be a kinetic barrier limited phase transformation process, and thus it could be thermally activated if enough energy is provided to overcome the barriers. The ligand design in quasi-2D perovskites offers us an opportunity to fine-tune the kinetic barrier by inhibiting the mass transport in the film. Specifically, our newly designed conjugated bulky ligand,

PPT' and PPT, are good at suppressing diffusion-limited phase disproportionation, leading to a relatively narrow *n*-phase distribution. PPT is slightly better than PPT' in terms of phase control behaviors since the increased dihedral angle by two methyl groups at 2- and 2'- positions would result in better solubility. This is expected to enhance the solution processability and film morphology, thus leading to better control over phase distribution.

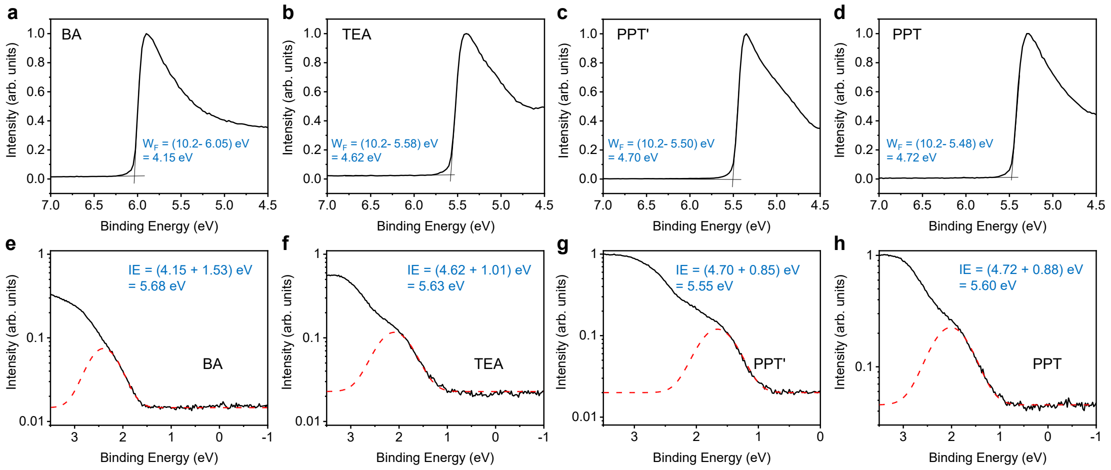

Supplementary Fig. 30 | Energy-level characterization of quasi-2D perovskite thin films. a-d, The secondary electron cut off region; **e-h,** The UPS spectra in the valence band region for (a,e) BA, (b,f) TEA, (c,g) PPT', (d,h) PPT films. Note: the UPS spectra were measured using H Lyman-α photon source with a photon energy of 10.2 eV and the Gaussian fit is used to determine the position of the valence band maximum (VBM) following previous work[7,8]. Specifically, the work function (W_F) for BA, TEA, PPT', and PPT samples are calculated to be 4.15, 4.62, 4.70, 4.72 eV, respectively. Based on onset from the gaussian fits, we can determine the ionization energy (IE) of BA, TEA, PPT', and PPT based quasi-2D perovskite thin films to be 5.68, 5.63, 5.55, 5.60 eV, respectively. The UPS signal comes primarily from the film surface (roughly the top 3 nm) and thus only reflects the phases that are present near the surface. If multiple phases are present, the ionization energy will represent that of the domains that are easiest to ionize (typically higher *n* values), at least assuming that there is a reasonable fraction (roughly 10% or more) of these domains present.

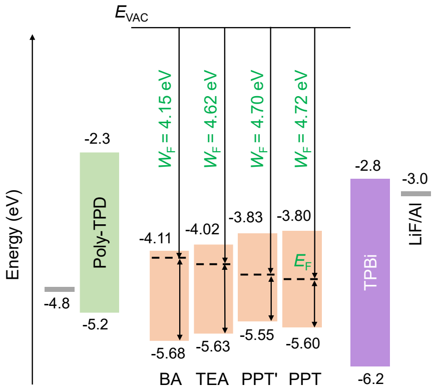

Supplementary Fig. 31 | Energy level diagram of quasi-2D perovskite LEDs. The energy levels of the electrodes (ITO and LiF/Al) and transporting materials (Poly-TPD and TPBi) are taken from literature[9]. The Fermi level and VBM of quasi-2D perovskite thin films are determined from the UPS measurement (Supplementary Fig. 30), respectively. The optical band gap derived from absorption spectra (Fig. 2a in the main text) was then used to estimate the conduction band minimum (CBM).

Both PPT' and PPT based 2D perovskites display greater work functions and slightly decreased ionization energies. The higher work functions of the PPT' and PPT sample relative to BA is attributed to the dipole of the aromatic ligands vs. BA as well as more n-type character for BA. In all films the surfaces are most likely terminated with organic ligands. In the case of the aromatic substituted ligands the aromatic units will withdraw electron density from the ethyl linker, resulting in a molecular dipole with a more electronegative tail group and thus a greater work function. The onset of the VB relative to the Fermi energy also decreases for the aromatic ligands relative to BA (Supplementary Fig. 31), which indicates that the BA films are more n-type and thus should display a decreased work function than the films with the aromatic ligands. The ionization energy changes are more subtle and may be attributed to small differences in the crystal structure (Pb-I bond angles), variations in phase distribution near the surface, and/or the influence of the aromatic units of ligand on stabilizing the hole generated during photoemission. For instance, regarding the phase distribution, BA and TEA films are dominated by 3D (n~∞) phase, which usually has a deep valance band level, indicating they may require a higher energy for ionization. While for PPT' and PPT cases, they are dominated by median-n (n~2-8) phase, which usually has a little bit shallow valance band, thus leading to slightly lower ionization energy. Based on the Fermi level, we noticed a clear transition from a heavily n-doped BA film to a near-intrinsic PPT film, which suggests a lower doping level in PPT film. This observation is consistent with PLQY measurements.

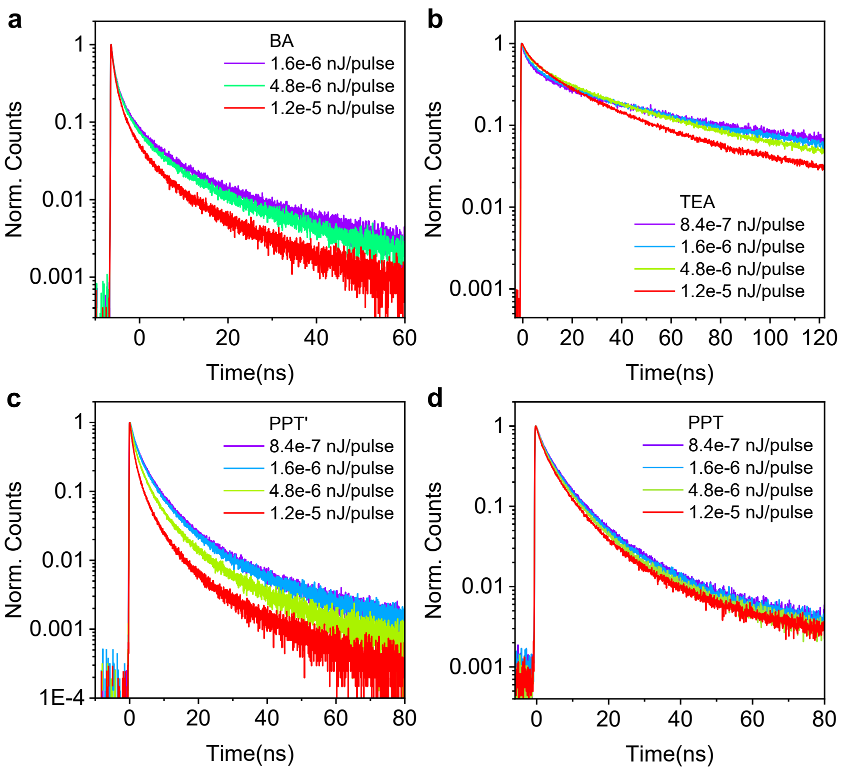

Supplementary Fig. 32 | Power dependent time-resolved photoluminescence (TRPL) study of quasi-2D perovskite thin films. a, BA; **b,** TEA; **c,** PPT'; **d,** PPT. All the BA, TEA and PPT' samples demonstrate faster PL decay with increasing excitation power, which is attributed to the higher order recombination process such as exciton-exciton annihilation or free carrier recombination in 3D phase. In addition, the TEA sample shows reduced fast decay component in PL lifetime with increasing excitation density, which is typically associated with trapping-detrapping process. In sharp contrast, the PPT film shows negligible power dependence in PL lifetime free from trap-filling, which indirectly confirms its excitonic nature as well as indicating that PPT film may have low defect densities with suppressed high-order exciton quenching[2].

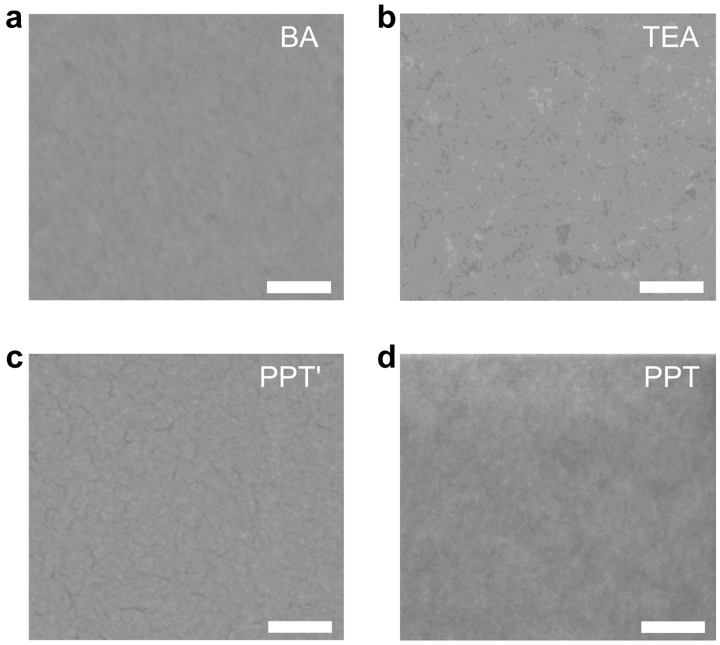

Supplementary Fig. 33 | SEM images of quasi-2D perovskite thin films fabricated from different organic ligands. a, BA; **b,** TEA; **c,** PPT'; **d,** PPT. Scale bars, 200 nm. All the SEM images show uniform films with complete surface coverage free of pinholes, which are crucial for reducing current leakage and balancing charge injection in LED devices.

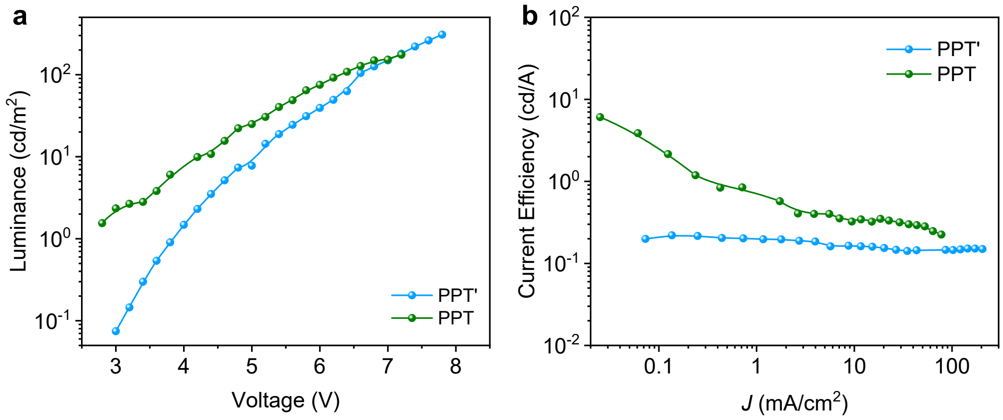

Supplementary Fig. 34 | LED device performances for PPT' and PPT devices. a, Luminance against voltage curves. **b,** Current efficiency plot against current density.

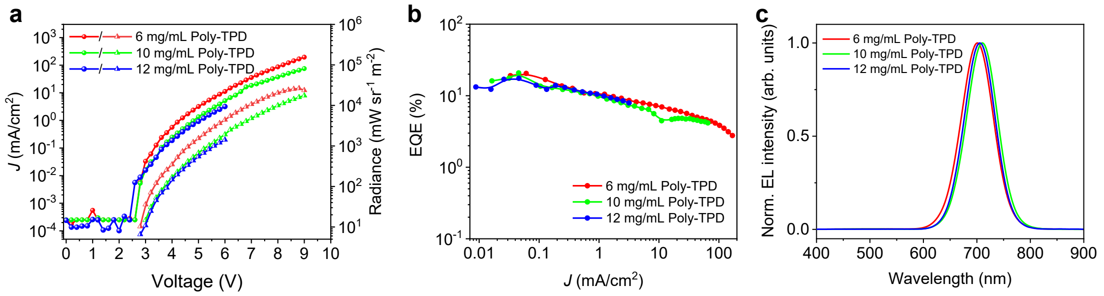

Supplementary Fig. 35 | Device characteristics of PPT perovskite LEDs based on hole transporting layer optimizations. a, Current density-radiance-voltage curves; **b,** the corresponding EQE-current density curves; and **c,** EL spectra of LED devices with different Poly-TPD thicknesses. The concentration of hole-transport materials (Poly-TPD) is optimized to be 8 mg/mL.

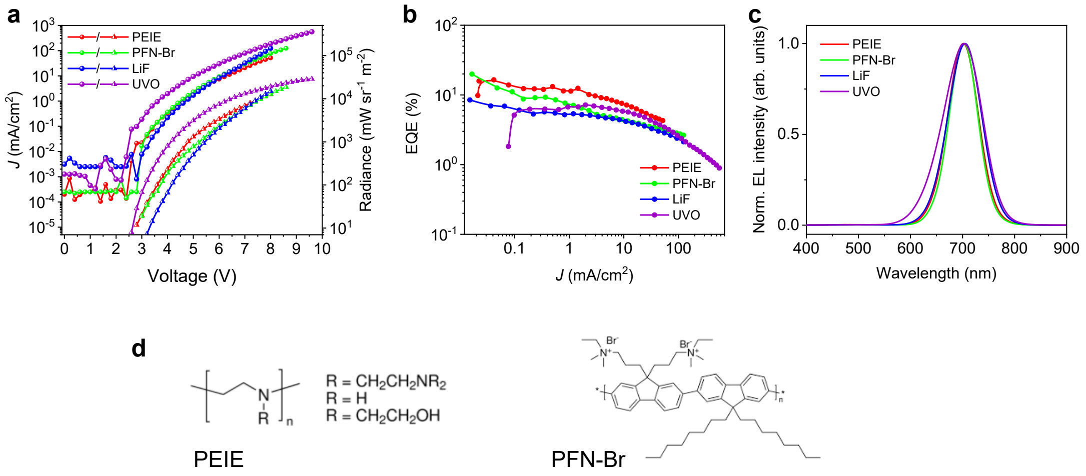

Supplementary Fig. 36 | Device characteristics of PPT perovskite LEDs based on interlayer treatment optimizations. a, Current density-radiance-voltage curves; **b,** the corresponding EQE-current density curves; and **c,** EL spectra of LED devices with different interlayer treatment. **d,** Chemical structures of two widely used polymer interlayer materials. Note, the concentration of PEIE and PFN-Br is 5 mg/mL, the thickness of LiF is 2 nm, and the UVO represents the Poly-TPD layer was treated by UV-ozone for 5 minutes before the deposition of perovskite layer.

Polymers are generally used as transporting layer in LED devices. Due to the hydrophobicity of polymer, the perovskite layer is hard to deposit onto the polymer surface directly with complete surface coverage. A variety of interfacial modification strategies have been proposed to improve the wettability of polymer, including UVO treatment, amphiphilic polymer interlayer, inorganic material layer, etc. As discussed in literature, UVO treatment will worsen the transporting behavior of the polymer transporting layer[10]. In our case, the device performance of the LED device based on UVO treatment did show the worst EQE efficiency. By incorporating inorganic interlayer (LiF), the device efficiency increased a little bit but rather limited. After introducing polymer interlayer, we can achieve highly efficient LEDs with EQE over 20%. The optimized interlayer is determined to be PVP.

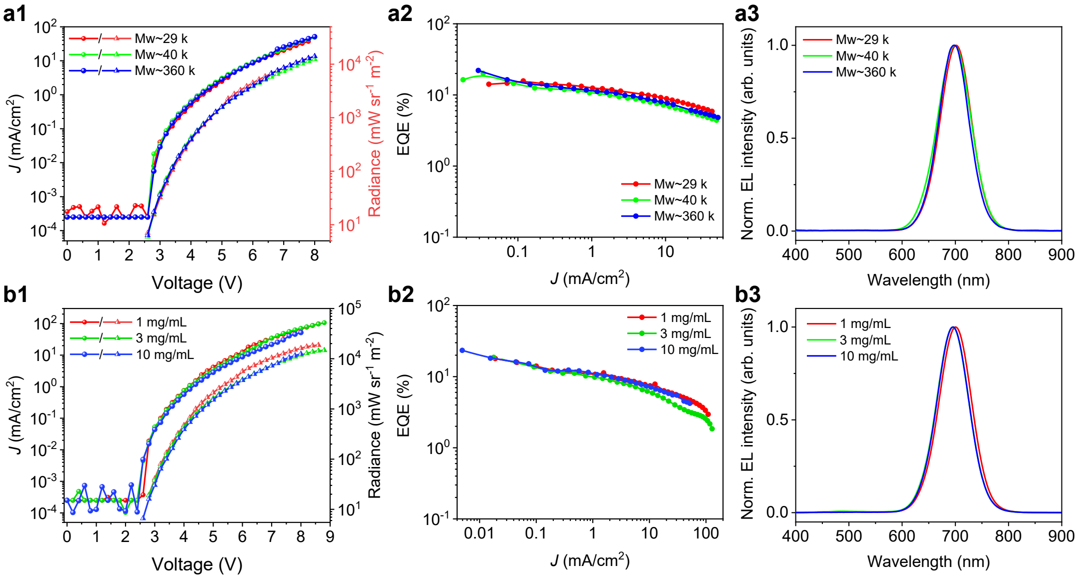

Supplementary Fig. 37 | Device characteristics of PPT perovskite LEDs based on PVP layer optimizations. a1,b1, Current density-radiance-voltage curves; **a2,b2,** the corresponding EQE-current density curves; and **a3,b3,** EL spectra of LED devices with different PVP molecular weight (a1-a3) and PVP precursor solution concentration (b1-b3). Based on the device results, the optimized PVP molecular weight is 1300 k and the PVP precursor solution concentration is optimized to be 5 mg/mL.

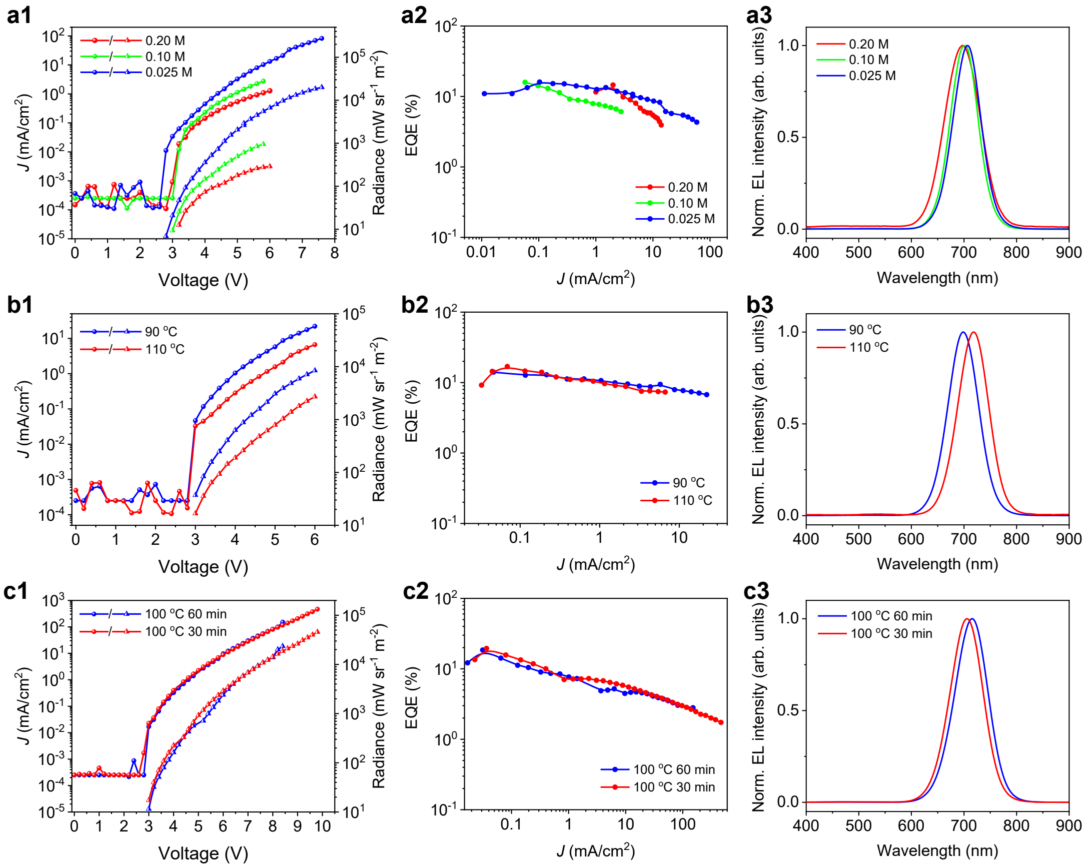

Supplementary Fig. 38 | Device characteristics of PPT perovskite LEDs based on perovskite layer optimizations. a1,b1,c1, Current density-radiance-voltage curves; **a2,b2,c2,** the corresponding EQE-current density curves; and **a3,b3,c3,** EL spectra of LED devices with different perovskite thicknesses (a1-a3), annealing temperatures (b1-b3), and annealing time (c1-c3). The EL wavelength shows a slight red shift after increasing the annealing temperature or extending the annealing time, which may suggest the further phase disproportionation into higher-*n* phase under higher temperature or longer time annealing. The optimized conditions for perovskite layer are with a concentration of 0.05 M annealed at 100 ºC for 10 min.

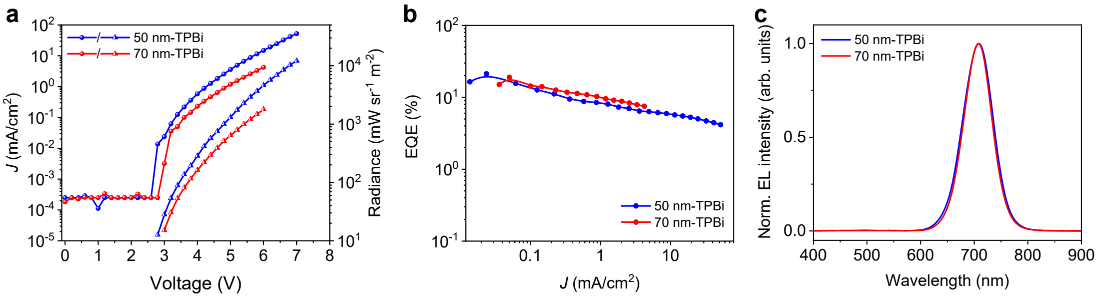

Supplementary Fig. 39 | Device characteristics of PPT perovskite LEDs based on electron transporting layer optimizations. a, Current density-radiance-voltage curves; **b,** the corresponding EQE-current density curves; and **c,** EL spectra of LED devices with different TPBi thicknesses. The optimized TPBi thickness is 60 nm.

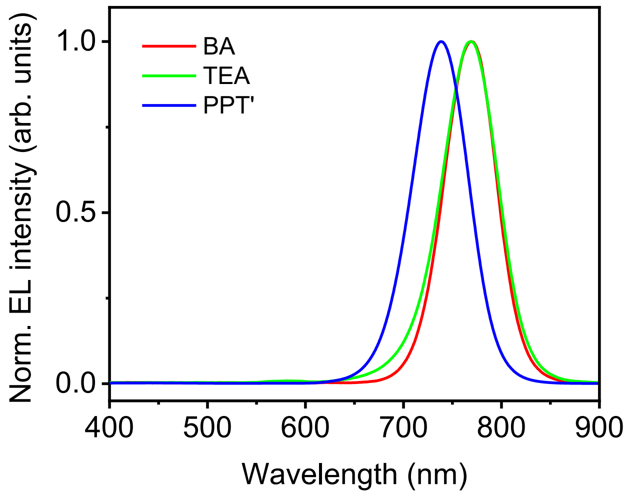

Supplementary Fig. 40 | EL spectra of quasi-2D perovskite LEDs based on other ligands of BA, TEA, and PPT'.

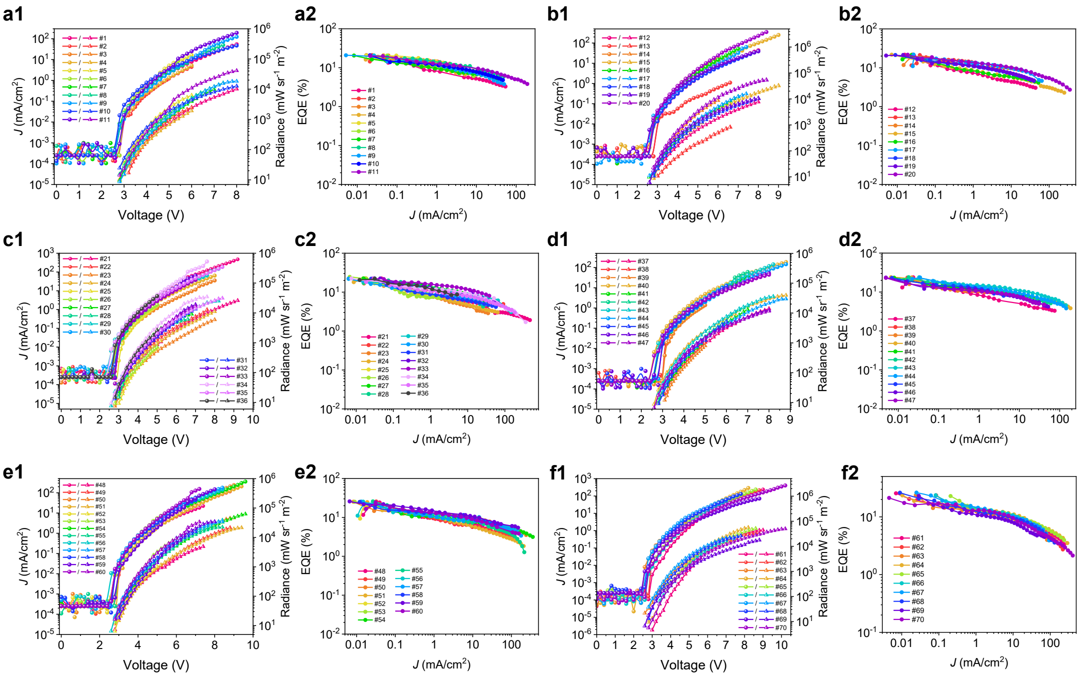

Supplementary Fig. 41 | Device characteristics for 70 devices used in EQE statistics in Fig. 4e in the main text. a1,b1,c1,d1,e1,f1, Current density-radiance-voltage curves. **a2,b2,c2,d2,e2,f2,** The corresponding EQE-current density curves.

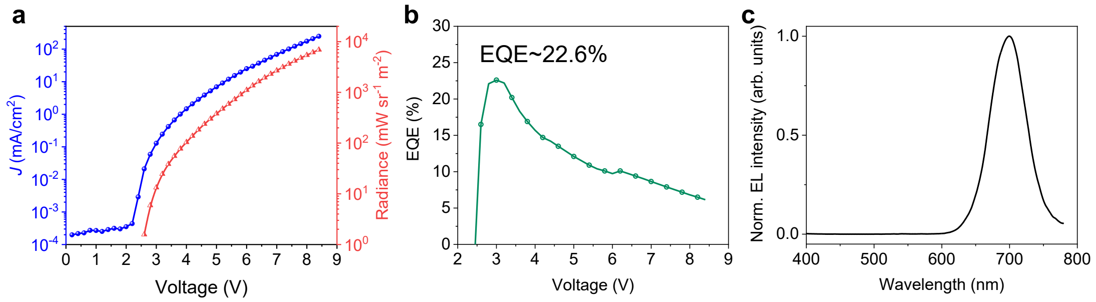

Supplementary Fig. 42 | Device performance of cross-checked PPT-based LEDs. a, Current density-radiance-voltage curves. **b,** the corresponding EQE-current density curves. **c,** Normalized EL spectra. The device was cross-checked at NCKU using PR655, which shows a peak EQE of 22.6% even after two-month international shipping delay because of the export control. The results are greatly consistent with that measured in our lab.

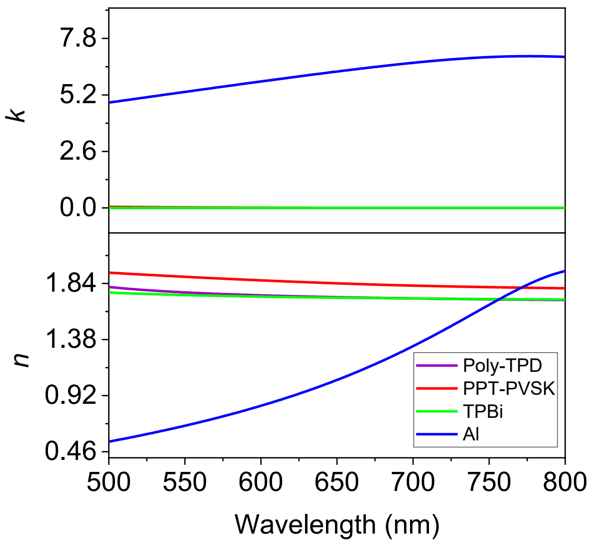

Supplementary Fig. 43 | Refractive indices of different layers in our perovskite LEDs. Optical constants (*n*, *k*) of the multilayers were retrieved from ellipsometry measurements. Here, the optical constants of perovskite layer are determined by using PPT ligand based quasi-2D perovskite (denote as PPT-PVSK) thin films. For Poly-TPD, PPT-PVSK, and TPBi, the extinction coefficient is near zero throughout the range of 500-800 nm. Specifically, both the refractive index of Poly-TPD and TPBi are measured to be 1.72 at 700 nm. Based on these optical constants, we obtained a 35.3% outcoupling efficiency for the champion device of PPT. This value is very close to the literature report by considering the similar device architecture, random dipole orientation, and similar refractive index of electron/hole transport materials[11].

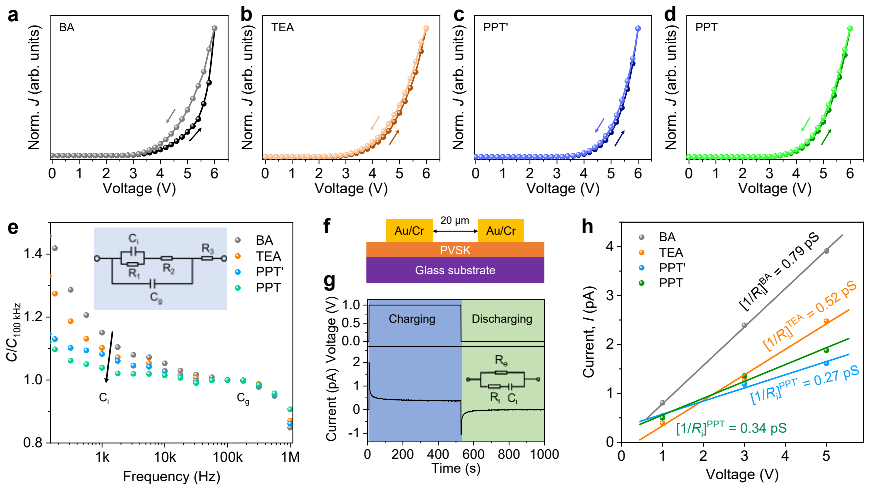

Supplementary Fig. 44 | Ionic transport related device physics. a-d, The *J-V* curves of LEDs based on (a) BA, (b) TEA, (c) PPT', (d) PPT, under forward and reverse scans. The scanning rate is 0.1 V per step and scanning range is 0 to 6 V for all measurements. The current density is normalized for comparison. **e,** The frequency dependent capacitance plots of LEDs based on different organic ligands with the equivalent circuit shown inset. C_g, geometric capacitance; C_i, mobile ion-dominated capacitance. The DC bias is set to zero and a small AC perturbation is set at 30 mV. **f-g,** Two-terminal device structure (f) for galvanostatic test by tracking the current decay during the charging and discharging process (g). The equivalent circuit is shown in the inset, where R_e is the electronic resistance, R_i is the ionic resistance, and C_i is the ionic capacitance. **h,** The relationship between ionic current and applied voltage, where the slop represents the ionic conductance. The ionic current was fitted from the fast decay component of the time-dependent current curve during charging.

BA-based device shows clear hysteresis loop in the forward and reverse *J-V* scans (Supplementary Fig. 44a), indicating the existence of significant ion migration. TEA and PPT' ligands can inhibit the ion migration under electric field, which leads to a reduced *J-V* loop (Supplementary Fig. 44b, c) in agreement with the results of thermally driven ion transport studies. More significantly, the forward and reverse *J-V* curves of PPT devices exhibit negligible hysteresis (Supplementary Fig. 44d), which suggests effective immobilization of ions in PPT devices.

Time-dependent electrical current was measured under constant voltage biases using two terminal devices with a channel width of 20 μm (Supplementary Fig. 44f). The results were analyzed using an equivalent circuit (Supplementary Fig. 44g, inset) which allowed us to differentiate electronic and ionic currents. The time-dependent current decay can be fitted well using a biexponential function in which the ionic

current is represented by the fast decay component. The slope of ionic current, as a function of the bias voltage, provides a measure of the ionic conductance (Supplementary Fig. 44h). Consistent with other theory and relevant measurements, the perovskites with BA and TEA indeed show a relatively higher ionic conductance of 0.79 and 0.52 pS, respectively; while the PPT' and PPT incorporated perovskites show a much lower value of 0.27 and 0.34 pS, respectively.

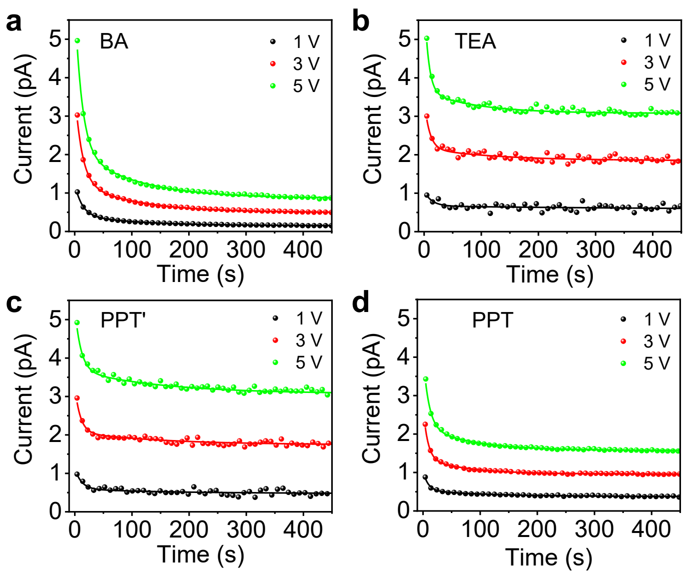

Supplementary Fig. 45 | Galvanostatic tests for quasi-2D perovskite thin films. a, BA; **b,** TEA; **c,** PPT'; **d,** PPT.

In the equivalent circuit shown in Supplementary Fig. 44g, the time-dependent current upon a turn-on of constant voltage can be expressed as

$$I(t) = \frac{V}{R_i} * exp\left(-\frac{t}{R_i C_i}\right) + \frac{V}{R_e} \tag{1}$$

where R_e is the electronic resistance, R_i is the ionic resistance, and C_i is the ionic capacitance. In addition, a slowly decaying component was noticed in the measured current, which can be related to irreversible material or interface degradations[12]. The total current was then assumed to follow a biexponential function:

$$I(t) = I_1 * exp\left(-\frac{t}{t_1}\right) + I_2 * exp\left(-\frac{t}{t_2}\right) + I_0 \tag{2}$$

The fast-decaying component $I_1 * exp\left(-\frac{t}{t_1}\right)$ corresponds to ionic current, whereas the constant term I_0 represents the electronic current. The coefficient I_1 was found to be linear with respect to the applied voltage, and the slope of I_1 against V can be considered as the ionic conductance. The fitted I_1 at different voltage can be found in Supplementary Table 4. In addition, the fitted time constant t_1 for the fast-decaying component is irrelevant of applied bias voltage (Supplementary Table 5), which demonstrates the reliability of our fitting process.

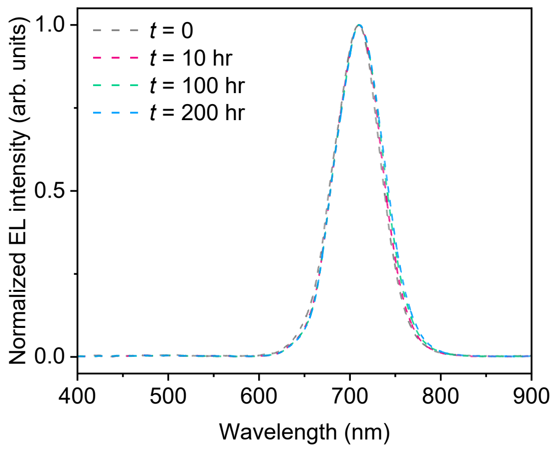

Supplementary Fig. 46 | Time evolution of EL spectra for PPT LED device during long-term stability tracking. The EL spectra did not show obvious shift during this long-term electrical operation, suggesting a good spectral stability in terms of suppressed ion diffusion and phase disproportionation.

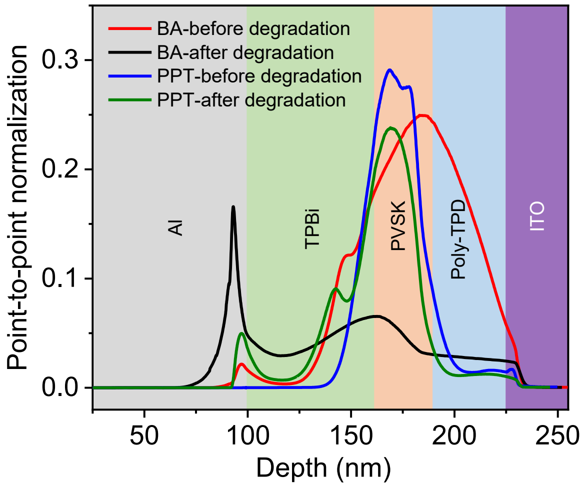

Supplementary Fig. 47 | Iodine profiles of the BA and PPT based devices before and after degradation obtained from ToF-SIMS measurements. The PPT-based devices exhibit significantly suppressed iodine diffusion compared with the BA-based devices. However, even though the iodide ions still diffuse into the TPBi layer after operating the device till degradation. This observation provides us a clue to further boost our device stability in the future by blocking the ion diffusion between perovskite and TPBi. Full elemental analysis can be found in Supplementary Fig. 48. Currently, the LED device's long-term stability is still a grand challenge. Besides suppressing ion migration, further investigation in terms of balancing charge injection, alleviating electrochemical reaction and joule heat effect may need to be conducted to boost the device stability to a next level.

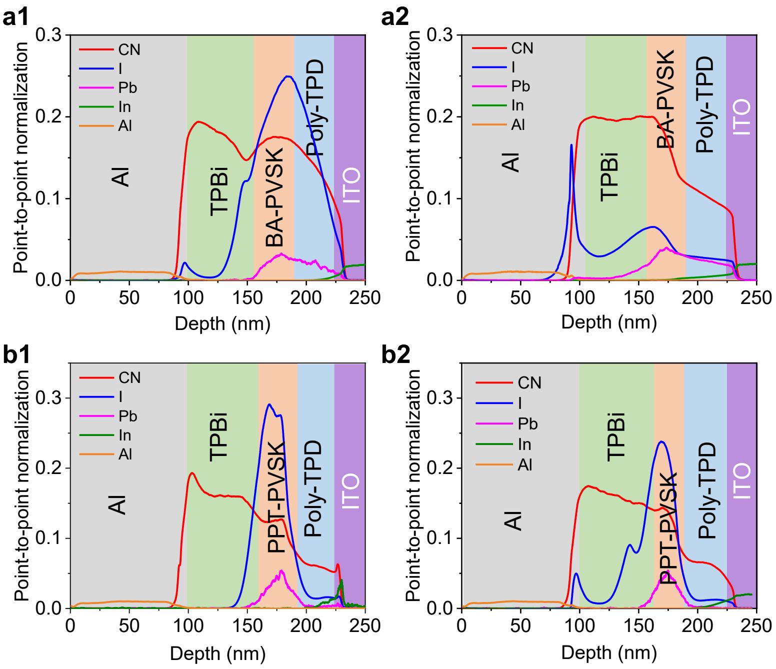

Supplementary Fig. 48 | Elemental distribution in quasi-2D perovskite LED devices based on ToF-SIMS measurements. a1,b1, Fresh; **a2,b2,** degraded devices for BA (a1 a2) and PPT (b1,b2) perovskites.

As ion migration is found to be a major cause of degradation for perovskite LED, we thus performed ToF-SIMS to examine the ion distribution in the degraded devices. A fresh device was used as a control. In the fresh device (Supplementary Fig. 48a1, b1), ToF-SIMS result shows a clear depth-dependent distribution profile for different materials, such as Al, CN, Pb, and I. Especially, the I and Pb show identical distribution, which indicates the location of perovskite layer. Nevertheless, BA-based device shows more significant accumulation of iodide ions at the interface of TPBi and Al than PPT-based device after degradation, which suggests severe iodide ion migration during device operation (Supplementary Fig. 48a2, b2). In addition, for BA based devices, the iodine diffuses extremely fast and deep into the TPBi layer even before applying bias; whereas PPT based devices exhibit slightly iodine diffusion only after device operation, suggesting that PPT ligand is better at blocking ion diffusion. Interestingly, the ion diffusion direction is opposite to the direction of electric field, which could be attributed to a concentration or thermal driven ion diffusion instead of electric field driven ion migration. We suppose that the electron transport layer (TPBi) is not dense enough, which is not able to block the ion diffusion effectively, leading to the ion accumulation close to the Al electrode and device degradation. Future work can be done to optimize the morphology of transporting layer to further suppress ion diffusion and enhance the device stability.

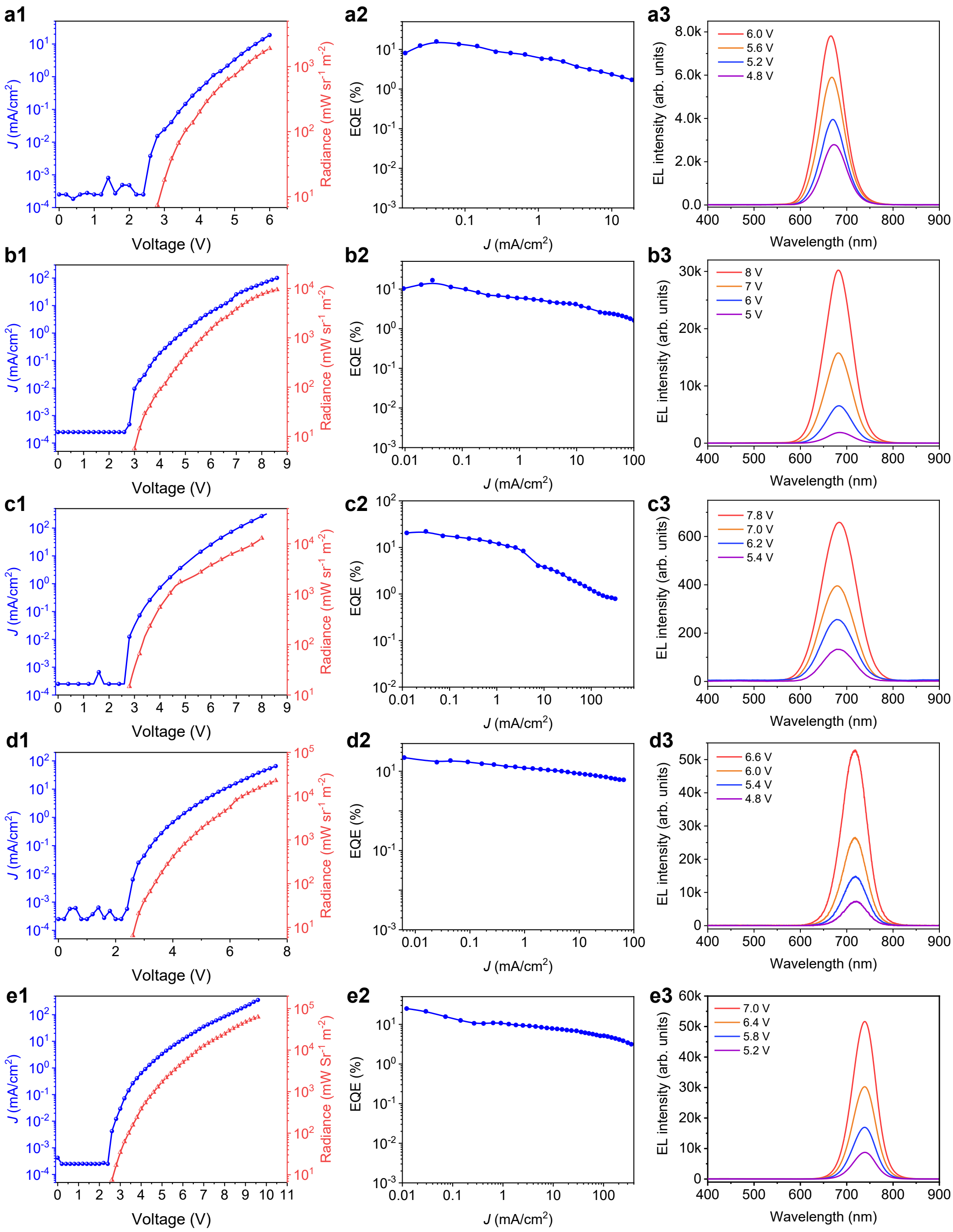

Supplementary Fig. 49 | Device characteristics of PPT perovskite LEDs with different EL peaks. a1,b1,c1,d1,e1, Current density-radiance-voltage curves. **a2,b2,c2,d2,e2,** The corresponding EQE-current density curves. **a3,b3,c3,d3,e3,** EL spectra under different driving voltages for peak at 666 (a1-a3), 679 (b1-b3), 684 (c1-c3), 721 (d1-d3), 744 nm (e1-e3). In all the devices, the EL peaks don't show obvious shift when increasing the driving voltage even to a very high level, which also indicates the suppression of ion diffusion under device operation condition.

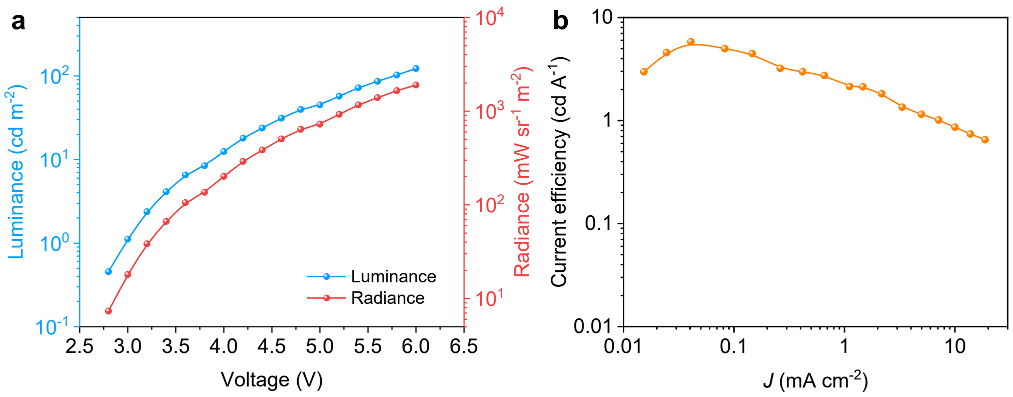

Supplementary Fig. 50 | Device characteristics of PPT perovskite LEDs with EL peaks at 666 nm ($Br_{0.2}I_{0.8}$). a, The luminance-radiance-voltage curves. **b,** The current efficiency plot against current density.

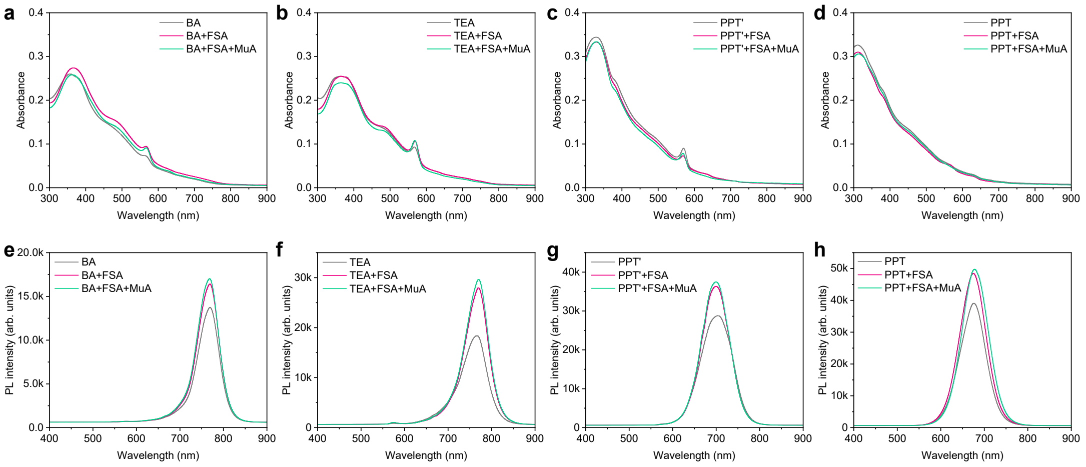

Supplementary Fig. 51 | Absorption (top) and PL (bottom) spectra of quasi-2D perovskite films after adding FSA and muconic acid (MuA). a,e, BA; **b,f,** TEA; **c,g,** PPT'; **d,h,** PPT.

FSA in the zwitterionic form is the most stable configuration in both the solid state and in perovskite precursor solution. Small amount of FSA has been reported to passivate both electron-donating (for example, FA vacancy) and electron-accepting (for example, halide vacancy, under-coordinated Pb^{2+}) defects on the perovskite grain surfaces[13]. Dicarboxylic group terminated molecules have been demonstrated to strongly interact with FAI and passivate the defect, and thus significantly boosting the LED device stability[14]. Here, FSA was added for all thin film and device studies, while muconic acid was introduced for device stability studies.

After adding the additives, such as FSA /and muconic acid, all films did not show obvious shift in their absorption and PL spectra, suggesting that the additives do not change the phase distribution too much since their doping ratios are relatively low. The PL enhancement after adding those dopants could be attributed to the defect passivation effect, which has been reported in previous literature. The introduction of muconic acid improves the device stability but does not lead to obvious device efficiency loss.

3. Supplementary tables and related discussions

Supplementary Table 1. Single crystal X-ray diffraction data of $(PPT)_2PbI_4$ and $(PPT')_2PbI_4$

	$(PPT)_2PbI_4$	$(PPT')_2PbI_4$
CCDC number	2113526	2166731
Crystal data		
Chemical formula	$2(C_{20}H_{22}NS)\cdot I_4Pb$	$I_{32}Pb_8$,16$(C_{20}H_{22}NS)$
*M*r	1331.68	10653.43
Crystal system, space group	monoclinic, *C 2/c*	Triclinic, P ī (2)
Temperature (K)	150	150
a, *b*, *c* (Å)	57.001(6), 6.2015(5), 12.2336(11)	12.158(3), 33.839(8), 42.511(9)
α, β, γ (°)	90, 99.269(4), 90	78.536(9), 85.959(12), 84.948(12)
V (Å^3)	4268.0(7)	17049
Z	4	2
F(000)	2496	9984
D_x (mg m^{-3})	2.072	2.075
Radiation type	Cu $K\alpha$	Cu $K\alpha$
No. of reflections for cell measurement	9773	9015
θ range (°) for cell measurement	3.1422-76.7396	5.2502-78.4840
μ (mm^{-1})	31.538	31.581
Crystal shape	Plate	Plate
Color	Yellow	Yellow
Crystal size (mm)	0.100 × 0.090 × 0.020	0.190 × 0.180 × 0.030
Data collection		
Diffractometer	Bruker AXS D8 Quest diffractometer with PhotonIII_C14 charge-integrating and photon counting pixel array detector	Bruker AXS D8 Quest diffractometer with PhotonIII_C14 charge-integrating and photon counting pixel array detector
Radiation source	I-μ-S microsource X-ray tube	I-μ-S microsource X-ray tube
Monochromator	Laterally graded multilayer (Goebel) mirror	Laterally graded multilayer (Goebel) mirror
Scan method	ω and ϕ scans	ω and ϕ scans
Absorption correction	Multi-scan<br>*SADABS* 2016/2: Krause, L., Herbst-Irmer, R., Sheldrick G.M. & Stalke D., J. Appl. Cryst. 48 (2015) 3-10	Multi-scan<br>*SADABS* 2016/2: Krause, L., Herbst-Irmer, R., Sheldrick G.M. & Stalke D., J. Appl. Cryst. 48 (2015) 3-10
T_{min}, T_{max}	0.055643, 0.178030	0.0271, 0.1427

No. of measured, independent and observed [$I > 2\sigma(I)$] reflections	4656, 4656, 3533	192522, 192522, 86218
R_{int}	0.0771	-
θ values (°)	θ_{max} = 79.758, θ_{min} = 3.142	θ_{max} = 79.196, θ_{min} = 2.124
$(\sin \theta/\lambda)_{max}$ (Å^{-1})	0.638	
Range of h, k, l	$-72 \leq h \leq 69$, $0 \leq k \leq 7$, $0 \leq h \leq 15$	$-15 \leq h \leq 12$, $-43 \leq k \leq 42$, $-53 \leq h \leq 53$
Refinement		
Refinement on	F^2	F^2
$R [F^2 > 2\sigma(F^2)]$, $wR(F^2)$, S	0.0952, 0.2317, 1.059	0.1521, 0.4308, 1.073
No. of reflections	4656	192522
No. of parameters	322	3363
No. of restraints	329	9499
H-atom treatment	H-atom parameters constrained	H-atom parameters constrained
Weighting scheme	$w = 1/[\sigma^2(F_o^2) + (0.1219P)^2 + 53.0058P]$ where $P = (F_o^2 + 2F_c^2)/3$	$w = 1/[\sigma^2(F_o^2) + (0.2284P)^2 + 55.9800P]$ where $P = (F_o^2 + 2F_c^2)/3$
$(\Delta/\sigma)_{max}$	0.000	0.002
$\Delta\rho_{max}$, $\Delta\rho_{min}$ (e Å^{-3})	3.166, -1.516	6.278, -5.155

Computer programs: Apex3 v2019.11-0 (Bruker, 2020), *SAINT V8.40B* (Bruker, 2020), *SHELXT* (Sheldrick, 2015), *SHELXL-2018/3* (Sheldrick, 2015, 2018), SHELXLE Rev1275 (Hübschle et al., 2011).

Single crystal structural details for $(PPT)_2PbI_4$:

The crystal under investigation was found to be non-merohedrally twinned. The orientation matrices for the two components were identified using the program Cell_Now, with the two components being related by a 180 degree rotation around the reciprocal axis (100). The two components were integrated using Saint and corrected for absorption using twinabs, resulting in the following statistics:

6842 data (1414 unique) involve domain 1 only, mean I/sigma 14.4
6647 data (1347 unique) involve domain 2 only, mean I/sigma 9.5
43896 data (4607 unique) involve 2 domains, mean I/sigma 18.8
92 data (78 unique) involve 3 domains, mean I/sigma 12.1

The exact twin matrix identified by the integration program was found to be:

0.99972 -0.02594 1.50842
-0.00030 -1.00000 -0.00074
0.00036 0.00201 -0.99972

The structure was solved using direct methods with only the non-overlapping reflections of component 1. The structure was refined using the hklf 5 routine with all reflections of component 1 (including the overlapping ones), resulting in a BASF value of 0.333(4).

The Rint value given is for all reflections and is based on agreement between observed single and composite intensities and those calculated from refined unique intensities and twin fractions (TWINABS (Sheldrick, 2012)).

The length of the b-axis is half the size of a chemical repeat unit of the Pb-I net, thus inducing systematic 1:1 disorder of the iodine atoms within the Pb-I layer (I1 and I2). The diffraction pattern was investigated for possible presence of satellite reflections along b and of a superstructure with double the volume, but no evidence was present. The lead ion is independently disordered across an inversion center.

The 1:1 iodine disorder induces disorder for the ethyl ammonium fragments (due to N-H···I hydrogen bonds). The ammonium and the first methylene group were included in the disorder. The two disordered moieties were restrained to have similar geometries. Uij components of ADPs for disordered atoms closer to each other than 2.0 Angstrom were restrained to be similar.

The outer tolyl group is disordered by rotation. The two disordered moieties were restrained to have similar geometries. The two moieties were each restrained to be close to planar. Uij components of ADPs for disordered atoms closer to each other than 2.0 Angstrom were restrained to be similar. Subject to these conditions the occupancy ratio refined to 0.666(14) to 0.334(14).

Single crystal structural details for $(PPT')_2PbI_4$:
The structure suffers from several types of non-merohedral twinning, leading to near to excessive overlap of a large fraction of reflections, preventing simultaneous integration of twin domains. The main type of twinning was found to be a rotation around the reciprocal (4 1 1) axis (twin transformation matrix 1 0 0, 0.5 -1 0, 0.5 0 -1).

Multiple other types of twinning appear to be present, but were not well enough resolved to be unambiguously assigned.

Attempts of simultaneous integration of the two main twin domains (related by the rotation around the reciprocal (4 1 1) axis proofed problematic due to excessive multiple overlap of reflections, resulting in large numbers of rejected reflections. Attempts were made to adjust integration parameters to avoid excessive rejections (through adjustments to integration queue size, integration box slicing and twin overlap parameters, and omission of box size optimization), which led to less but still substantial numbers of rejected reflections. Attempts at refinement against these incomplete and biased data gave unsatisfactory results (R1 > 25%, wR2 > 70%).

With no complete data set obtainable through simultaneous integration of both twin domains, the data were instead handled as if not twinned, with only the major domain integrated, and converted into an hklf 5 type format hkl file after integration using the "Make HKLF5 File" routine as implemented in WinGX. The twin law matrix was used as obtained from SAINT, see above. The Overlap R1 and R2 values used were 0.25, i.e. reflections with a discriminator function less or equal to overlap radius of 0.25 were counted overlapped, all others as single. The discriminator function used was the "delta function on index non-integrality". No reflections were omitted.

The structure was solved using dual methods (ShelXT) with the original hklf 4 type file and was refined using the hklf 5 type file created via WinGX, resulting in a BASF value of 0.407(3).

Omission of twinning results in apparent disorder for the iodine atoms in each of the Pb-planes (I1, I3, I8, I10, etc) and apparent disorder of Pb ions (with minor Pb ions being bonded to the other terminal iodine atoms). Residual electron densities associated with this apparent disorder are between 8 and 12 electrons per cubic Angstrom (double the values for the largest difference densities with twinning included).

No Rint value is obtainable for the hklf 5 type file using the WinGX routine. The value from the original HKLF 4 type file is given instead.

The structure is eight-fold modulated, with a modulation function being close to a slightly distorted sin/cosine curve, which is most clearly pronounced for the lead and

iodine atoms. Pb and I atoms of a chain stretching along the [0 1 1] diagonal of the unit cell follow a “wavy line” and are related by pseudo-translations along that direction (positions of Pb and I atoms are freely refined without use of restraints or constraints). Modulation is broken by the orientation of the CH_2-CH_2-NH_3 groups, which are the same every four of the eight repeat units. The four-fold repeat is not quite commensurate with the positions of the Pb and I atoms, breaking exact translational symmetry after four repeat units. The remainder of the ligands follow mostly a two-fold repeat, with the ligands being subject to a pseudo-glide plane operation (glide direction is the [0 1 1] modulation direction, the mirror component is parallel to (0 1 1). a slight variation of torsion angles of the middle phenylene ring is observed, further breaking exact two-, four or eight-fold translation.

Modulation leads to a large number of weak diffraction spots (around 50% < 2 sigma for all resolution shells), and only around 45% of diffraction spots are > 2 sigma ("observed").

Attempts to refine the data is smaller unit cells based on the pseudo-translations closest to actual translations led to Pb and I atoms with extremely elongated ADPs and substantially increased R values.

The additional insufficiently resolved twinning that was necessarily ignored leads to large numbers of outlier reflections with I(obs) >> I(calc), and the presence of substantial residual electron densities (up to 6.3 electrons per cubic Angstrom). The positions of large residual electron densities are in agreement with Pb, I and S atoms shifted 1.5 to 2 Angstrom along the modulation direction. Attempts were made to refine these as disordered moieties to ensure that the residuals do indeed result from unresolved twinning. Results were far from satisfactory with at least three-fold disorder required to account for all large residual peaks and insufficient decrease of R value to justify inclusion of disorder.

Due to the intrinsically low quality of the data at hand a number of restraints were included in the structure refinement. The geometries of all sixteen independent organic ligands were restrained to be similar to each other (SAME restraint). In the initial refinement, the ADPs of C, N, and S atoms related by close to exact translation were constrained to be identical, and components of ADPs of C, N, and S atoms closer to each other than 2 Angstrom were restrained to be similar to each other (SIMU 0.01). After model stabilization the SIMU restrained was replaced by a RIGU

restraint (esd 0.001 Angstrom squared), and EADP constraints were removed for most atoms (they were retained for the atoms of the CH_2-CH_2-NH_3 groups). A mild anti-bumping restraint was used to avoid close contacts of methyl H atoms.

Supplementary Table 2. Performance summary of quasi-2D perovskite LEDs recently reported. (Note: For a fair comparison of device stability, we suggest fully considering wavelength, efficiency, current density, and initial brightness rather than only paying attention to T_{50} numbers)

Emitter	EL wavelength /Color	Peak EQE (%)	T_{50} (h)	Lifetime test conditions	References
PEABr-CsPb($Cl_{0.9}Br_{2.1}$)	480 nm/Blue	5.7	0.17	4.4 V	Yip et al[15]
$PBABr_y(Cs_{0.7}FA_{0.3}PbBr_3)$	483 nm/Blue	9.5	0.07	100 cd/m^2 @ 1 mA/cm^2	Jin et al[16]
$PEA_2Cs_{n-1}Pb_nBr_{3n+1}$-GABA	~480 nm/Blue	6.3	0.03	~150 cd/m^2	Sargent et al[17]
$PEA_2Cs_{1.6}MA_{0.4}Pb_3Br_{10}$-DPPOCl	479 nm/Blue	5.2	1.5	100 cd/m^2	Sargent et al[18]
$CsPbBr_3$-PEACl-YCl_3	485 nm/Sky-blue	11.0	1.7	100 cd/m^2	Huang et al[19]
$CsPbClBr_2$-DPPABr-PEABr	473 nm/Blue	8.8	0.1	100 cd/m^2 @ 1.95 mA/cm^2	Zhong et al[20]
$PEA_2(CsPbBr_3)_2PbBr_4$-EABr	488 nm/Sky-Blue	12.1	0.06	100 cd/m^2 @ 1.5 mA/cm^2	You et al[21]
PFNBr-PEABr-CsBr-$PbBr_2$-$PbCl_2$	485 nm/Blue	11.2	0.20	530 cd/m^2 @ 5 mA/cm^2	Liao et al[22]
$(PEOA)_2(MA)_{n-1}Pb_nBr_{3n+1}$	520 nm/Green	2.82	N.A.	N.A.	Yip et al[23]
$PEA_2(FAPbBr_3)_{n-1}PbBr_4$	532 nm/Green	14.36	1.2	125 cd/m^2 @ 0.3 mA/cm^2	You et al[24]
PEABr-$CsPbBr_3$-crown	515 nm/Green	15.5	1.5	2 mA/cm^2	Sun et al[25]
PEABr-$FAPbBr_3$	527 nm/Green	12.4	1.5	100 cd/m^2 @ 1 mA/cm^2	Adachi et al[26]
$PEA_2Cs_{2.4}MA_{0.6}Pb_4Br_{13}$-TPPO	~520 nm/Green	14.0	0.7	4000 cd/m^2	Sargent et al[27]
PBABr-PABr-$FAPbBr_3$	534 nm/Green	15.1	N.A.	N.A.	Su et al[28]
PEABr-$CsPbBr_3$-TPPCl	520 nm/Green	19.1	N.A.	N.A.	Friend et al[10]
$BA_2Cs_{n-1}Pb_nBr_{3n+1}$-MeS	512 nm/Green	20.5	0.38	1000 cd/m^2	Yang et al[29]
$PEA_2(CsPbBr_3)_4PbBr_4$-ETPTA	508 nm/Green	22.49	0.83	144 cd/m^2 @ 0.3 mA/cm^2	You et al[30]
$FA_{0.7}Cs_{0.3}PbBr_3$-PEABr-NVAL	520 nm/Green	21.3	1.95	100 cd/m^2	Yuan et al[31]
PEABr-$(Cs_{0.8}MA_{0.2})PbBr_3$-TFPPO	510 nm/Green	25.6	1.92	7200 cd/m^2 @ 8 mA/cm^2	Sargent et al[32]
$CsPbI_3$-BAI-PEO	680 nm/Red	6.23	~4	3.5 V	Ma et al[33]
$PBA_2Cs_{n-1}Pb_nI_{3n+1}$	665 nm/Red	13.3	0.003	110 cd/m^2	Jin et al[34]
$PEA_2MA_2Pb_3I_{10-x}Cl_x$	650 nm/Red	2.2	1.45	100 mA/cm^2	Li et al[35]
$(mFPEA_xNMA_{1-x})_2CsPb_2I_7$	680 nm/Red	25.8	0.6	100 cd/m^2	You et al[66]
$(PEA)_2(MA)_{n-1}Pb_nI_{3n+1}$	760 nm/NIR	8.8	N.A.	N.A.	Sargent et al[36]
$(NMA)_2(FAPbI_3)PbI_4$	760 nm/NIR	11.7	~1.2	10 mA/cm^2	Huang et al[37]

$(NMA)_2(FA)_{n-1}Pb_nI_{3n+1}$	~800 nm/NIR	20.1	46	0.1 mA/cm^2	Friend et al[38]
$(PVBA)_2(FA_{0.83}Cs_{0.17})_9Pb_{10}I_{31}$	750 nm/NIR	23.2	20.8*	5 mA/cm^2	Xiao et al[39]
**$(PPT)_2(FAPbBr_{0.6}I_{2.4})_2PbI_4$**	**~684 nm/Red**	**22.1**	**220<br>30.9<br>7.1<br>2.8**	**0.1 mA/cm^2<br>1 mA/cm^2<br>5 mA/cm^2<br>12 mA/cm^2@100 cd/m^2**	**This work**
**$(PPT)_2(FAPbI_3)_2PbI_4$**	**~700 nm/Deep red**	**26.3**			
**$(PPT)_2(FAPbI_3)_3PbI_4$**	**~721 nm/Deep red**	**22.1**			

*This stability data was measured from n = 2 based devices instead of n = 10 based champion devices.

Supplementary Table 3. Wavelength tunable LEDs based on controllable *n*-distribution quasi-2D perovskite thin films with different precursor solutions. Specifically, <*n*3> represents that the stoichiometry ratio of precursor solution follows the general chemical formula of $(PPT)_2FA_{n-1}Pb_nI_{3n+1}$ with a nominal <*n*> = 3.

Emitter	Precursor solution	Concentration, Pb^{2+} (mM)	Ratio	Peak EQE (%)	EL peak (nm)
$[Br_{0.2}I_{0.8}]^{<n>=3}$	$(PPT)_2PbI_4$:$FAPbI_3$:$FAPbBr_3$		1:1.2:0.8	15.9	666
$[I]^{<n>=2}$	$(PPT)_2PbI_4$:$FAPbI_3$		1:1	16.7	679
$[Br_{0.1}I_{0.9}]^{<n>=3}$	$(PPT)_2PbI_4$:$FAPbI_3$:$FAPbBr_3$	50	1:1.6:0.4	22.1	684
$[I]^{<n>=3}$	$(PPT)_2PbI_4$:$FAPbI_3$		1:2	26.3	701
$[I]^{<n>=4}$	$(PPT)_2PbI_4$:$FAPbI_3$		1:3	22.1	721
$[I]^{<n>=5}$	$(PPT)_2PbI_4$:$FAPbI_3$		1:4	21.3	740

Supplementary Table 4. Fitted weighing coefficients for fast-decaying components during charging process.

I_1 (pA)	BA	TEA	PPT'	PPT
**1 V**	0.807 ± 0.008	0.390 ± 0.065	0.535 ± 0.056	0.501 ± 0.016
**3 V**	2.392 ± 0.018	1.275 ± 0.099	1.189 ± 0.063	1.357 ± 0.020
**5 V**	3.908 ± 0.031	2.476 ± 0.145	1.612 ± 0.061	1.877 ± 0.024

Supplementary Table 5. Fitted time constants for fast-decaying components during charging process.

t_1 (s)	BA	TEA	PPT'	PPT
**1 V**	12.602 ± 0.216	13.473 ± 3.166	11.661 ± 1.893	8.876 ± 0.393
**3 V**	12.276 ± 0.164	9.102 ± 0.914	9.504 ± 0.708	7.961 ± 0.173
**5 V**	12.290 ± 0.171	7.250 ± 0.489	10.412 ± 0.582	9.933 ± 0.197

4. ^{1}H, ^{13}C NMR and HR-MS spectra

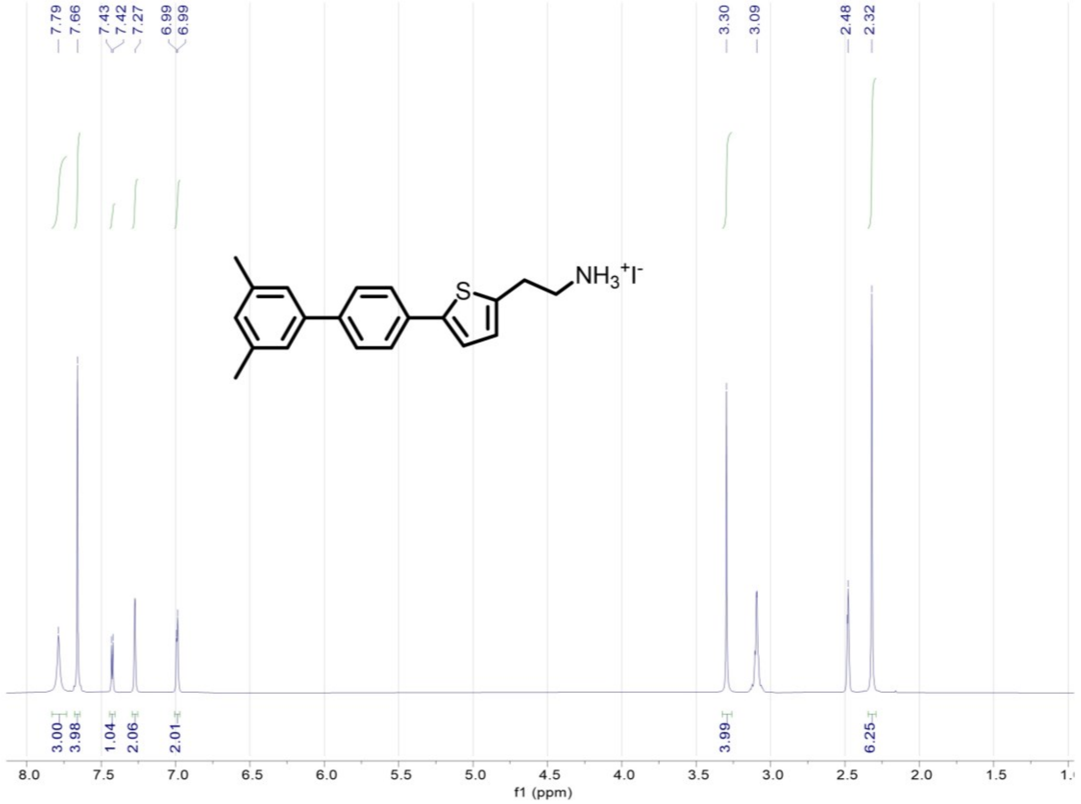

^{1}H NMR spectrum of **PPT'** in DMSO-d_6

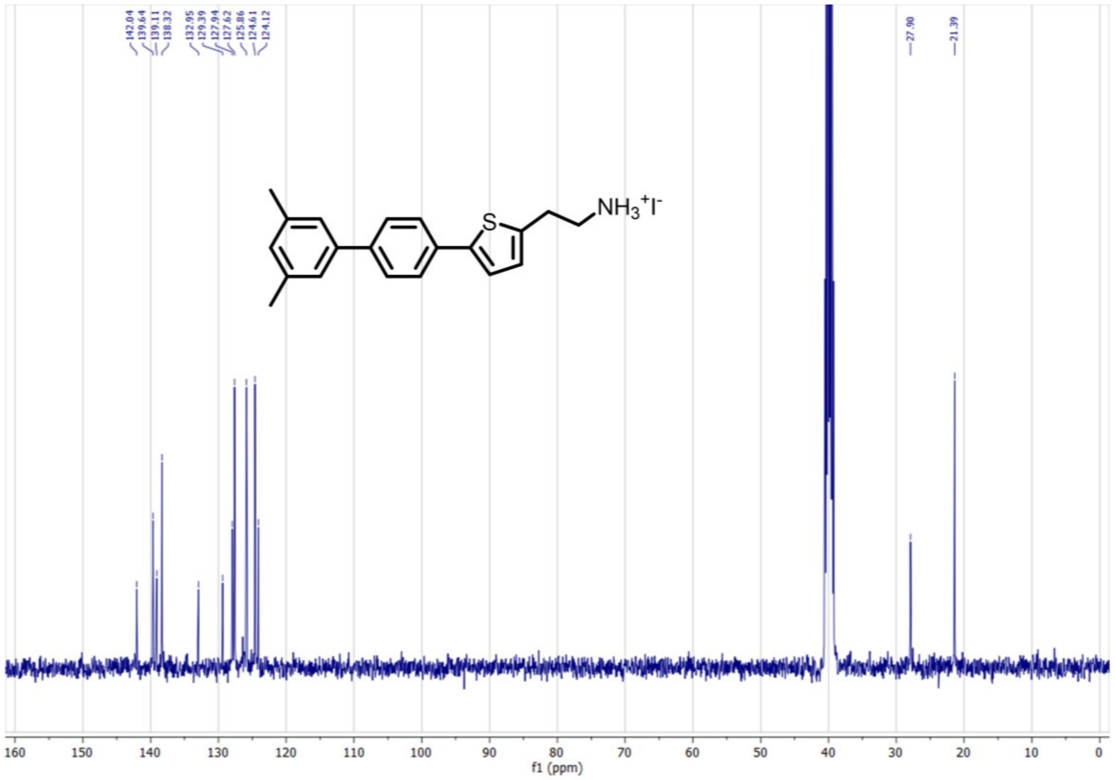

^{13}C NMR spectrum of **PPT'** in DMSO-d_6

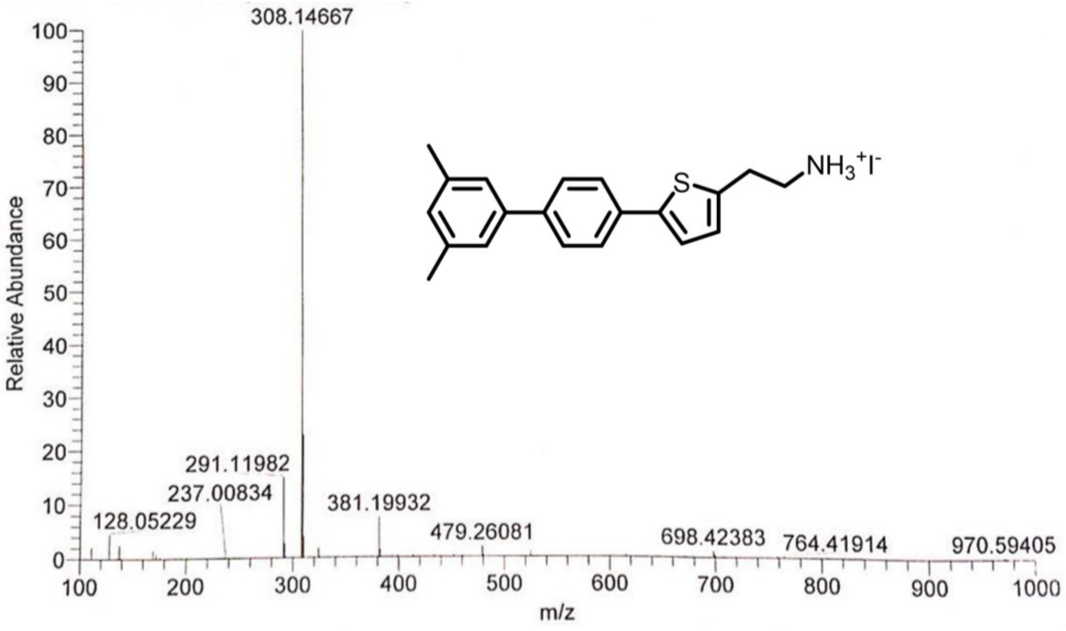

High resolution mass spectrometry (HR-MS, ESI) of **PPT'**.

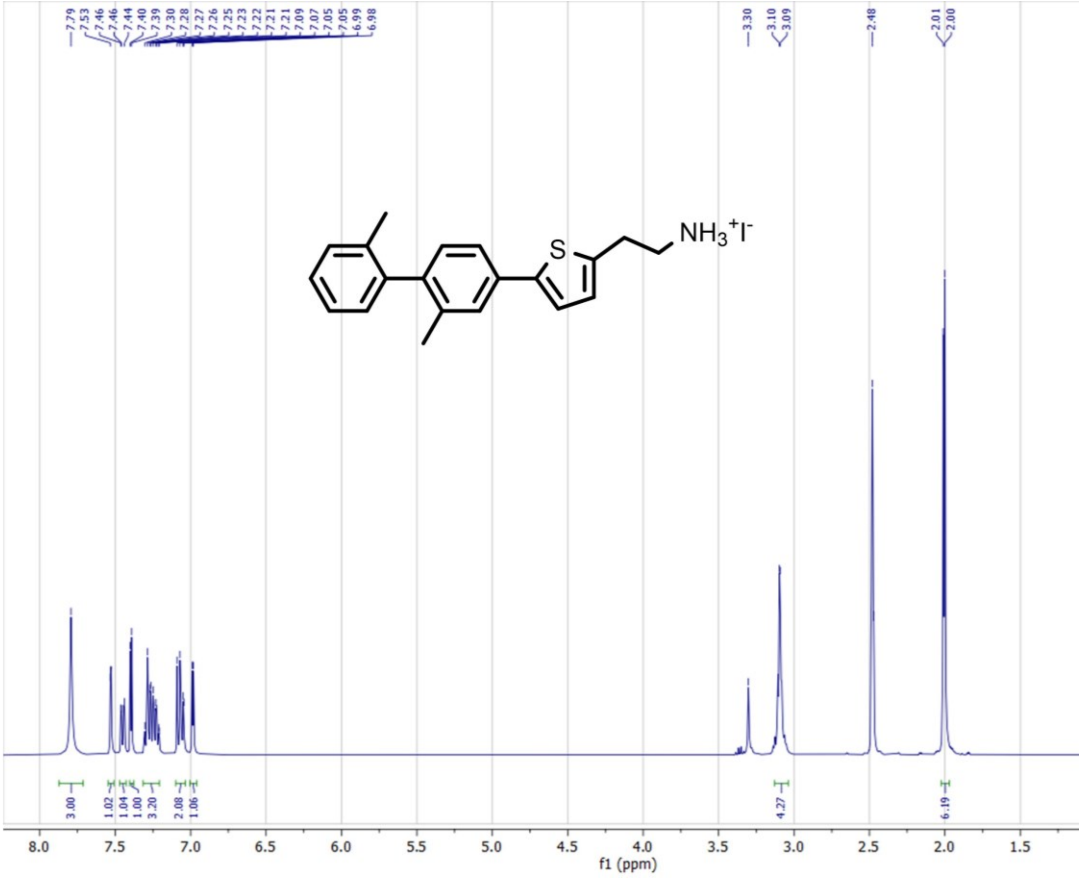

^{1}H NMR spectrum of **PPT** in DMSO-d_6

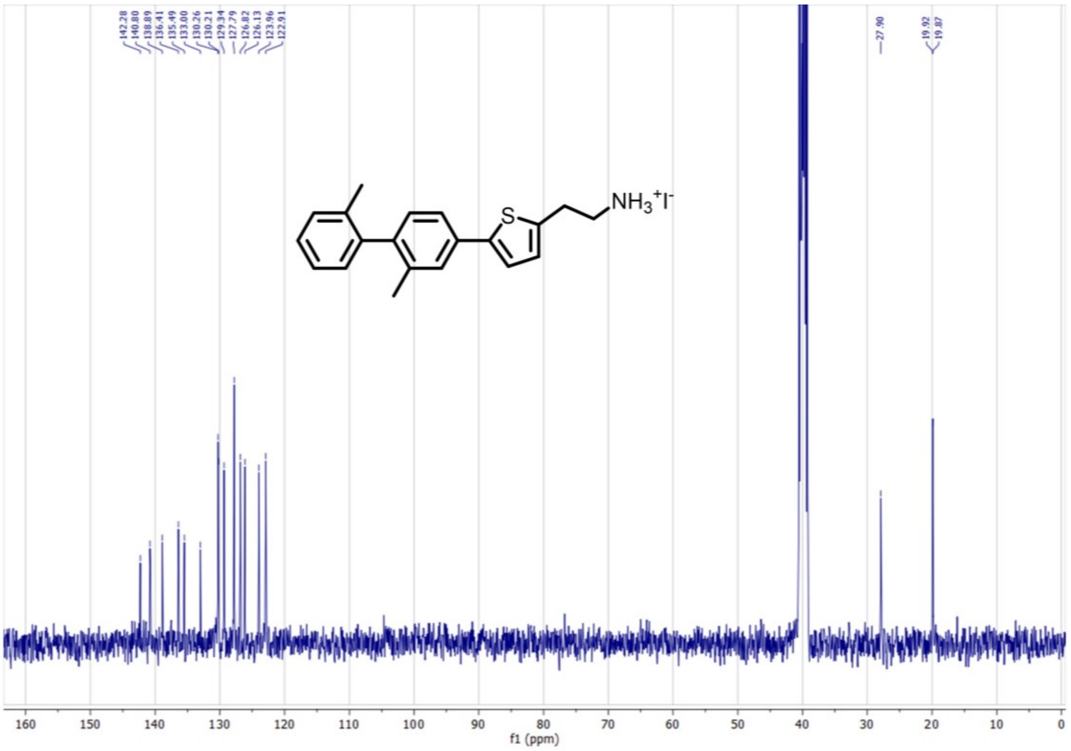

^{13}C NMR spectrum of **PPT** in DMSO-d_6

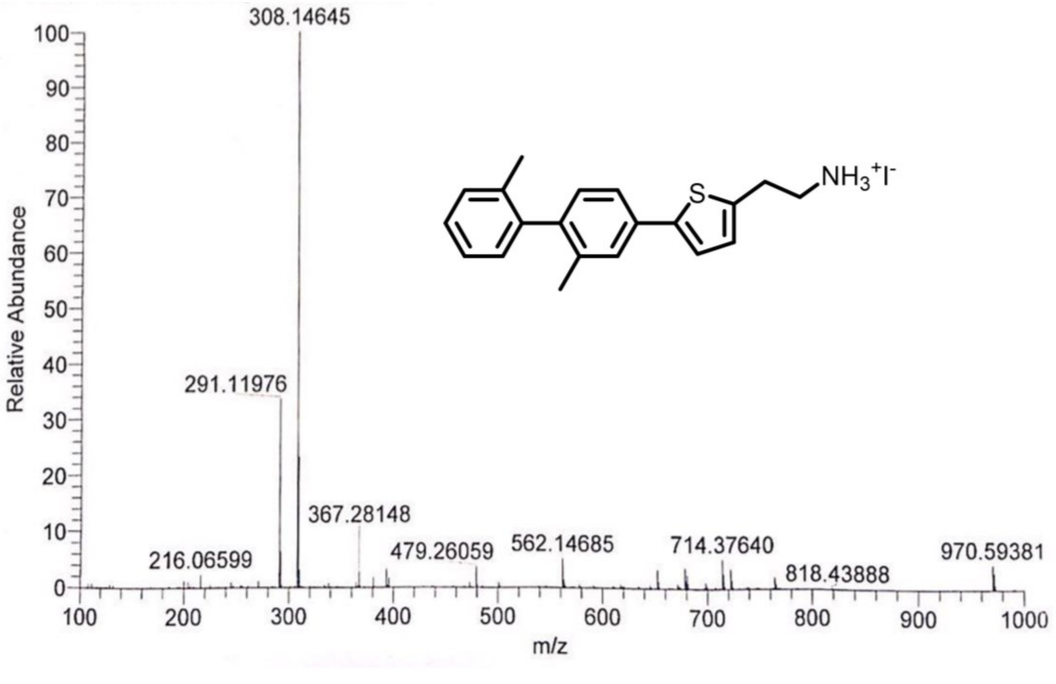

High resolution mass spectrometry (HR-MS, ESI) of **PPT**.

Supplementary References

1 Gao, Y. *et al.* Molecular engineering of organic–inorganic hybrid perovskites quantum wells. *Nat. Chem.* **11**, 1151-1157 (2019).

2 Deng, S. *et al.* Long-range exciton transport and slow annihilation in two-dimensional hybrid perovskites. *Nat. Commun.* **11**, 664 (2020).

3 Leguy, A. M. A. *et al.* Reversible hydration of $CH_3NH_3PbI_3$ in films, single crystals, and solar cells. *Chem. Mater.* **27**, 3397-3407 (2015).

4 Cao, Y. *et al.* Perovskite light-emitting diodes based on spontaneously formed submicrometre-scale structures. *Nature* **562**, 249-253 (2018).

5 Moore, D. T. *et al.* Crystallization kinetics of organic-inorganic trihalide perovskites and the role of the lead anion in crystal growth. *J. Am. Chem. Soc.* **137**, 2350-2358 (2015).

6 Yi, C. *et al.* Intermediate-phase-assisted low-temperature formation of γ-$CsPbI_3$ films for high-efficiency deep-red light-emitting devices. *Nat. Commun.* **11**, 4736 (2020).

7 Endres, J. *et al.* Electronic structure of the $CsPbBr_3$/polytriarylamine (PTAA) system. *J. Appl. Phys.* **121**, 035304 (2017).

8 Boehm, A. M., Liu, T., Park, S. M., Abtahi, A. & Graham, K. R. Influence of surface ligands on energetics at $FASnI_3$/C60 interfaces and their impact on photovoltaic performance. *ACS Appl. Mater. Interfaces* **12**, 5209-5218 (2020).

9 Hassan, Y. *et al.* Ligand-engineered bandgap stability in mixed-halide perovskite LEDs. *Nature* **591**, 72-77 (2021).

10 Zhao, B. *et al.* Efficient light-emitting diodes from mixed-dimensional perovskites on a fluoride interface. Nat. *Electron.* **3**, 704-710 (2020).

11 Shin, H. *et al.* Sky-blue phosphorescent OLEDs with 34.1% external quantum efficiency using a low refractive index electron transporting layer. *Adv. Mater.* **28**, 4920-4925 (2016).

12 Domanski, K. *et al.* Migration of cations induces reversible performance losses over day/night cycling in perovskite solar cells. *Energy Environ. Sci.* **10**, 604-613 (2017).

13 Xiao, K. *et al.* All-perovskite tandem solar cells with 24.2% certified efficiency and area over 1 cm^2 using surface-anchoring zwitterionic antioxidant. *Nat. Energy* **5**, 870-880 (2020).

14 Kuang, C. *et al.* Critical role of additive-induced molecular interaction on the operational stability of perovskite light-emitting diodes. *Joule* **5**, 618-630 (2021).

15 Li, Z. *et al.* Modulation of recombination zone position for quasi-two-dimensional blue perovskite light-emitting diodes with efficiency exceeding 5%. *Nat. Commun.* **10**, 1027 (2019).

16 Liu, Y. *et al.* Efficient blue light-emitting diodes based on quantum-confined bromide perovskite nanostructures. Nat. *Photonics* **13**, 760-764 (2019).

17 Wang, Y.-K. *et al.* Chelating-agent-assisted control of $CsPbBr_3$ quantum well growth enables stable blue perovskite emitters. Nat. *Commun.* **11**, 3674 (2020).

18 Ma, D. *et al.* Chloride insertion-immobilization enables bright, narrowband, and stable blue-emitting perovskite diodes. *J. Am. Chem. Soc.* **142**, 5126-5134 (2020).

19 Wang, Q. *et al.* Efficient sky-blue perovskite light-emitting diodes via photoluminescence enhancement. *Nat.* Commun. **10**, 5633 (2019).

20 Wang, C. *et al.* Dimension control of in situ fabricated $CsPbClBr_2$ nanocrystal films toward efficient blue light-emitting diodes. *Nat. Commun.* **11**, 6428 (2020).

21 Chu, Z. *et al.* Large cation ethylammonium incorporated perovskite for efficient and spectra stable blue light-emitting diodes. *Nat. Commun.* **11**, 4165 (2020).

22 Yuan, S. *et al.* Efficient and spectrally stable blue perovskite light-emitting diodes employing a cationic π-conjugated polymer. *Adv. Mater.* **33**, 2103640 (2021).

23 Chen, Z. *et al.* High-performance color-tunable perovskite light emitting devices through structural modulation from bulk to layered film. *Adv. Mater.* **29**, 1603157 (2017).

24 Yang, X. *et al.* Efficient green light-emitting diodes based on quasi-two-dimensional composition and phase engineered perovskite with surface passivation. *Nat. Commun.* **9**, 570 (2018).

25 Ban, M. *et al.* Solution-processed perovskite light emitting diodes with efficiency exceeding 15% through additive-controlled nanostructure tailoring. *Nat. Commun.* **9**, 3892 (2018).

26 Qin, C. *et al.* Triplet management for efficient perovskite light-emitting diodes. *Nat. Photonics* **14**, 70-75 (2019).

27 Quan, L. N. *et al.* Edge stabilization in reduced-dimensional perovskites. *Nat. Commun.* **11**, 170 (2020).

28 Meng, F. *et al.* Co-interlayer engineering toward efficient green quasi-two-dimensional perovskite light-emitting diodes. *Adv. Funct. Mater.* **30**, 1910167 (2020).

29 Kong, L. *et al.* Smoothing the energy transfer pathway in quasi-2D perovskite films using methanesulfonate leads to highly efficient light-emitting devices. *Nat. Commun.* **12**, 1246 (2021).

30 Chu, Z. *et al.* Perovskite light-emitting diodes with external quantum efficiency exceeding 22% via small-molecule passivation. *Adv. Mater.* **33**, e2007169 (2021).

31 Sun, C. *et al.* High-performance large-area quasi-2D perovskite light-emitting diodes. *Nat. Commun.* **12**, 2207 (2021).

32 Ma, D. *et al.* Distribution control enables efficient reduced-dimensional perovskite LEDs. *Nature* **599**, 594-598 (2021).

33 Tian, Y. *et al.* Highly efficient spectrally stable red perovskite light-emitting diodes. *Adv. Mater.* **30**, 1707093 (2018).

34 He, Z. *et al.* High-efficiency red light-emitting diodes based on multiple quantum wells of phenylbutylammonium-cesium lead iodide perovskites. *ACS Photonics* **6**, 587-594 (2019).

35 Deng, L. *et al.* Effective phase control for high-performance red-light-emitting quasi-2D perovskite solar cells via MACl additive. *ACS Appl. Energy Mater.* **4**, 2856-2863 (2021).

36 Yuan, M. *et al.* Perovskite energy funnels for efficient light-emitting diodes. *Nat. Nanotechnol.* **11**, 872 (2016).

37 Wang, N. *et al.* Perovskite light-emitting diodes based on solution-processed self-organized multiple quantum wells. *Nat. Photonics* **10**, 699 (2016).

38 Zhao, B. *et al.* High-efficiency perovskite-polymer bulk heterostructure light-emitting diodes. *Nat. Photonics* **12**, 783-789 (2018).

39 Chen, W. *et al.* Polymerized hybrid perovskites with enhanced stability, flexibility, and lattice rigidity. *Adv. Mater.* **33**, 2104842 (2021).
